# Disproportionate impacts of COVID-19 in a large US city

**DOI:** 10.1371/journal.pcbi.1011149

**Published:** 2023-06-01

**Authors:** Spencer J. Fox, Emily Javan, Remy Pasco, Graham C. Gibson, Briana Betke, José L. Herrera-Diestra, Spencer Woody, Kelly Pierce, Kaitlyn E. Johnson, Maureen Johnson-León, Michael Lachmann, Lauren Ancel Meyers

**Affiliations:** 1 Department of Epidemiology & Biostatistics, University of Georgia, Athens, Georgia, United States of America; 2 Institute of Bioinformatics, University of Georgia, Athens, Georgia, United States of America; 3 Center for the Ecology of Infectious Diseases, University of Georgia, Athens, Georgia, United States of America; 4 Department of Integrative Biology, The University of Texas at Austin, Austin, Texas, United States of America; 5 Department of Industrial Engineering, The University of Texas at Austin, Austin, Texas, United States of America; 6 Los Alamos National Laboratory, Los Alamos, New Mexico, United States of America; 7 The Texas Advanced Computing Center, The University of Texas at Austin, Austin, Texas, United States of America; 8 The Rockefeller Foundation, New York, New York, United States of America; 9 The Santa Fe Institute, Santa Fe, New Mexico, United States of America; Fundação Getúlio Vargas: Fundacao Getulio Vargas, BRAZIL

## Abstract

COVID-19 has disproportionately impacted individuals depending on where they live and work, and based on their race, ethnicity, and socioeconomic status. Studies have documented catastrophic disparities at critical points throughout the pandemic, but have not yet systematically tracked their severity through time. Using anonymized hospitalization data from March 11, 2020 to June 1, 2021 and fine-grain infection hospitalization rates, we estimate the time-varying burden of COVID-19 by age group and ZIP code in Austin, Texas. During this 15-month period, we estimate an overall 23.7% (95% CrI: 22.5–24.8%) infection rate and 29.4% (95% CrI: 28.0–31.0%) case reporting rate. Individuals over 65 were less likely to be infected than younger age groups (11.2% [95% CrI: 10.3–12.0%] vs 25.1% [95% CrI: 23.7–26.4%]), but more likely to be hospitalized (1,965 per 100,000 vs 376 per 100,000) and have their infections reported (53% [95% CrI: 49–57%] vs 28% [95% CrI: 27–30%]). We used a mixed effect poisson regression model to estimate disparities in infection and reporting rates as a function of social vulnerability. We compared ZIP codes ranking in the 75th percentile of vulnerability to those in the 25th percentile, and found that the more vulnerable communities had 2.5 (95% CrI: 2.0–3.0) times the infection rate and only 70% (95% CrI: 60%-82%) the reporting rate compared to the less vulnerable communities. Inequality persisted but declined significantly over the 15-month study period. Our results suggest that further public health efforts are needed to mitigate local COVID-19 disparities and that the CDC’s social vulnerability index may serve as a reliable predictor of risk on a local scale when surveillance data are limited.

## Author summary

COVID-19 disproportionately impacted communities based on their socioeconomic and racial composition. Studies have documented catastrophic disparities at multiple geographic scales, but have not yet tracked how they evolved over time. Here, we use fine-grain epidemiological data to estimate the time-varying disparate burden of COVID-19 by age group and ZIP code in Austin, Texas. During this 15-month period, we estimate that 23.7% (95% CrI: 22.5–24.8%) of the population was infected and 29.4% (95% CrI: 28.0–31.0%) of those infections were reported. Infections were not spread evenly across the region. Individuals over 65 were significantly less likely to be infected than younger age groups (11.2% [95% CrI: 10.3–12.0%] vs 25.1% [95% CrI: 23.7–26.4%]), suggesting that efforts to protect those populations may have been effective. We found that the most vulnerable ZIP codes in the region faced 2.5 times the infection risks compared with the least vulnerable ZIP codes, and that infections were only 70% as likely to be reported in vulnerable communities. Inequality persisted but declined significantly over the 15-month study period. Further public health efforts are needed to address local COVID-19 disparities.

## Introduction

The WHO estimates that the COVID-19 pandemic caused nearly 15 million excess deaths worldwide between its emergence in 2019 and the end of 2021. The burden fell disproportionately on countries in South-East Asia, Europe, and the Americas, with 68% of the estimated excess deaths occurring in 10 countries containing 35% of the global population [1]. In the United States, pandemic burden was initially concentrated around New York City, New York, but spread geographically after the White House issued the *Opening Up America Again* guidelines in spring of 2020 [2]. The pandemic disproportionately harmed essential workers and racial and ethnic minority groups [3–6] as well as US counties [7–10] and cities [11–13] with high social vulnerability indices [14].

In response to these glaring disparities, scientists and public health leaders advocated for programs to support marginalized communities, including accessible testing facilities, community support programs to mitigate the socioeconomic, educational and healthcare harms resulting from lockdowns, proactive vaccination and antiviral campaigns, and effective public health communications [15–21]. Many US vaccination campaigns successfully prioritized vulnerable regions [22–29], though others, such as in Texas, limited geographic prioritization efforts [30,31].

To prevent, detect, and reduce disparities in infectious disease burden, we need to increase the geographic and temporal resolution of our surveillance efforts, while reducing biases. Published estimates of COVID-19 burden in underserved populations are often derived directly from reported case or death counts, without correcting for ascertainment biases or disentangling risks of infection from risks of severe outcomes [7–9,32–42]. When available, both serological [43,44] and hospitalization data [45] can be used to estimate reporting rates. Several studies have highlighted the disproportionate burden of COVID-19 infections within cities [46,47], but only at single time points during the pandemic.

Here, we estimate the changing burden of COVID-19 at a local scale within a large US city throughout the first 15 months of the pandemic. Using ZIP-code and age-stratified hospitalization data, we track daily disparities in infection rates, hospitalization rates, and case reporting rates. As the SARS-CoV-2 virus continues to evolve along with our arsenal of medical and behavioral interventions, this method can help to ensure the reliability and equity of local risk assessments [48].

## Results

**Fig 1:**
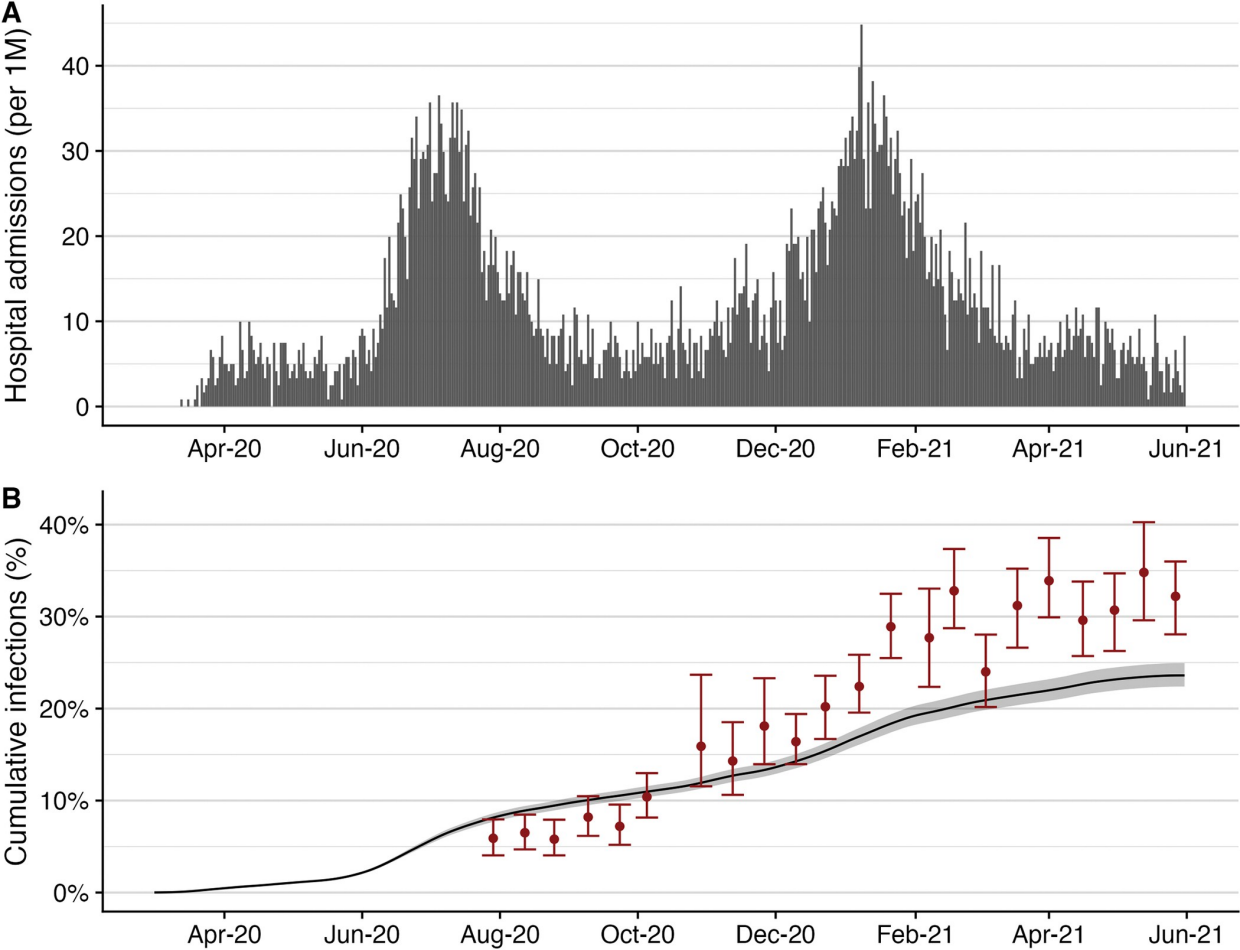
COVID-19 hospital admissions and estimated cumulative infections for Travis County (Austin, TX) from March 1, 2020 to June 1, 2021. (A) Daily reported COVID-19 hospital admissions per 1 million residents [50]. (B) Estimated cumulative infections with 95% credible intervals (black line and gray ribbon) compared to Texas statewide seroprevalence-based estimates (red points and error bars) [49].

We analyzed spatial COVID-19 burdens in Austin, Texas using hospital admission data from March 11, 2020 to June 1, 2021. This period preceded the emergence of the Delta variant and included a small wave in April 2020, followed by larger waves in the summer and winter (Figs 1A and S1). As of June 1, 2021, there were 83,722 reported cases, 6,474 hospitalized patients, and 1,024 deaths of COVID-19 in Travis County, which has 1.3 million residents, covering 57% of the Austin metropolitan area population. We estimate that 23.7% (95% CrI: 22.5–24.8%) of the population were infected in this time period and 29.4% (95% CrI: 28.0–31.0%) of all infections were reported. Statewide seroprevalence data suggest that Texans were 1.3 times as likely to be infected in this time period, with an estimated attack rate of 32% (95% CrI: 28–36%) (Fig 1B) [49]. However, the estimated infection risks prior to September 23, 2020 are higher for Travis county (10.6% [95% CrI: 10.0–11.1%] infected) than statewide (7.2% [95% CrI: 5.2–9.6%] infected).

**Fig 2:**
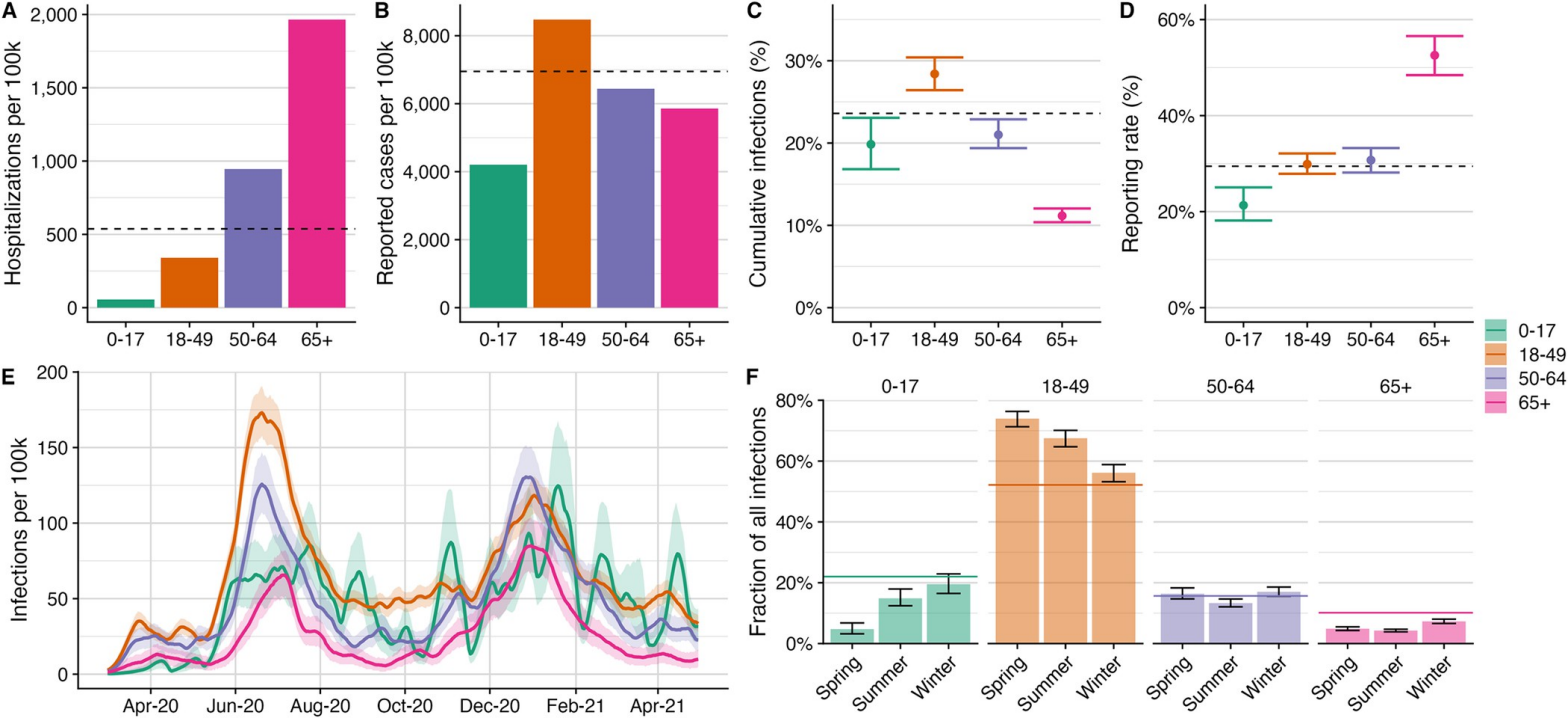
Estimated age-stratified COVID-19 burden in Travis country through June 1, 2021. (A) Reported COVID-19 hospital admissions by age group. (B) Reported COVID-19 cases by age group. (C) Estimated percent infected by age group. (D) Estimated COVID-19 case reporting rates by age group up to June 1, 2021. In (A)-(D), horizontal dashed lines indicate county-wide average rates. (E) Estimated daily infection rates (line) and 95% credible intervals (ribbons) by age group. (F) Distribution of infections across age groups for each period of the epidemic. The spring period refers to the two-month time period before the first major wave from March 1, 2020 to May 1, 2020, the summer period refers to the two-month period containing the first major wave from June 1, 2020 to August 1, 2020, and the winter period refers to the two-month period containing the second major wave from December 1, 2020 until February 1, 2021. Bars indicate the fraction of all infections during the time period in each age group, with the error bars indicating the 95% credible intervals. The horizontal colored lines in panel F indicate the proportion of the Travis county population in the specified age group.

In Travis County, children aged 0–17 experienced the lowest hospitalization risk, with a cumulative count of 55.8 hospital admissions per 100,000, and adults over age 65 experienced the highest hospitalization risk of 1,965 per 100,000 (Fig 2A). In contrast, reported cases were relatively similar across age groups, ranging from 4,206 per 100,000 in children to 8,475 per 100,000 in young adults (Fig 2B). Using age-specific seroprevalence and hospital admissions data for the state of Texas, we estimate that one in 434 (95% CI: 243–625) infections in individuals aged 0–17 years and one in 4.7 (95% CI: 3.0–6.8) infections in individuals over age 65 led to hospitalization (Table 1). This is consistent with published estimates for the infection hospitalization rate from China [51] and France [45] (S2 Fig).

**Table 1:** SARS-CoV-2 infection hospitalization rate (IHR) across Texas estimated from statewide seroprevalence and hospitalization data from July 29, 2020 through May 27, 2021.

Age group	Number of COVID-19 hospital admissions (95% CI) [52]	Estimated infections based on seroprevalence data (95% CI) [53,54]	Estimated IHR (95% CI)
0–17	4,870 (4,727–4,975)	2,081,849 (1,195,168–2,972,145)	0.25% (0.16%-0.41%)
18–49	53,913 (53,106–54,597)	3,795,068 (2,827,349–4,773,427)	1.45% (1.13%-1.90%)
50–64	58,466 (57,114–59,473)	1,084,729 (718,420–1,481,055)	5.57% (3.96%-8.16%)
65+	100,881 (98,032–102,931)	499,962 (303,726–692,758)	21.12% (14.54%-33.04%)

By June 1, 2021, we estimate that 28.5% (95% CrI: 26.6–30.5%) of 18–49 year olds were infected, while only 11.2% (95% CrI: 10.3–12.0%) of individuals over age 65 were infected (Fig 2C). The estimated percent of cases reported increases with age, ranging from 21.3% (95% CrI: 18.2–24.8%) in 0–17 year olds to 52.6% (95% CrI: 48.7–56.9%) in over 65 year olds (Fig 2D). All age groups experienced two large waves during the study period, though the summer 2020 was relatively mild for children (Fig 2E). Relative infection rates across age groups evened out over time (Fig 2F). For example, children, who account for 22% of the Travis county population, constituted 4.8% (95% CrI: 3.2–6.9%) of all infections between March 1, 2020 and May 1, 2020 and 19.6% (95% CrI: 16.5–22.9%) of all infections between December 1, 2020 and February 1, 2021. The proportion of infections occurring in 18–49 year olds, who make up 52.2% of the population, dropped from 73.9% (95% CrI: 71.3–76.5%) during the spring 2020 period to 56.1% (95% CrI: 53.3–59.0%) during the winter 2020–2021 wave (Figs 2F and S3). Reported case and hospitalization counts do not clearly exhibit this reversal in age-specific risks (S4 and S5 Figs). Infection rates for each age group were estimated to be lower in Travis county than statewide, by factors of 44% (95% CrI: 20–60%), 19.5% (95% CrI: 0.2–34%), 23.1% (0.2–40%), and 28.6% (95% CrI: 0.03–47%) in the 0–17, 18–49, 50–64, and 65+ year groups, respectively (S6 and S7 Fig).

**Fig 3:**
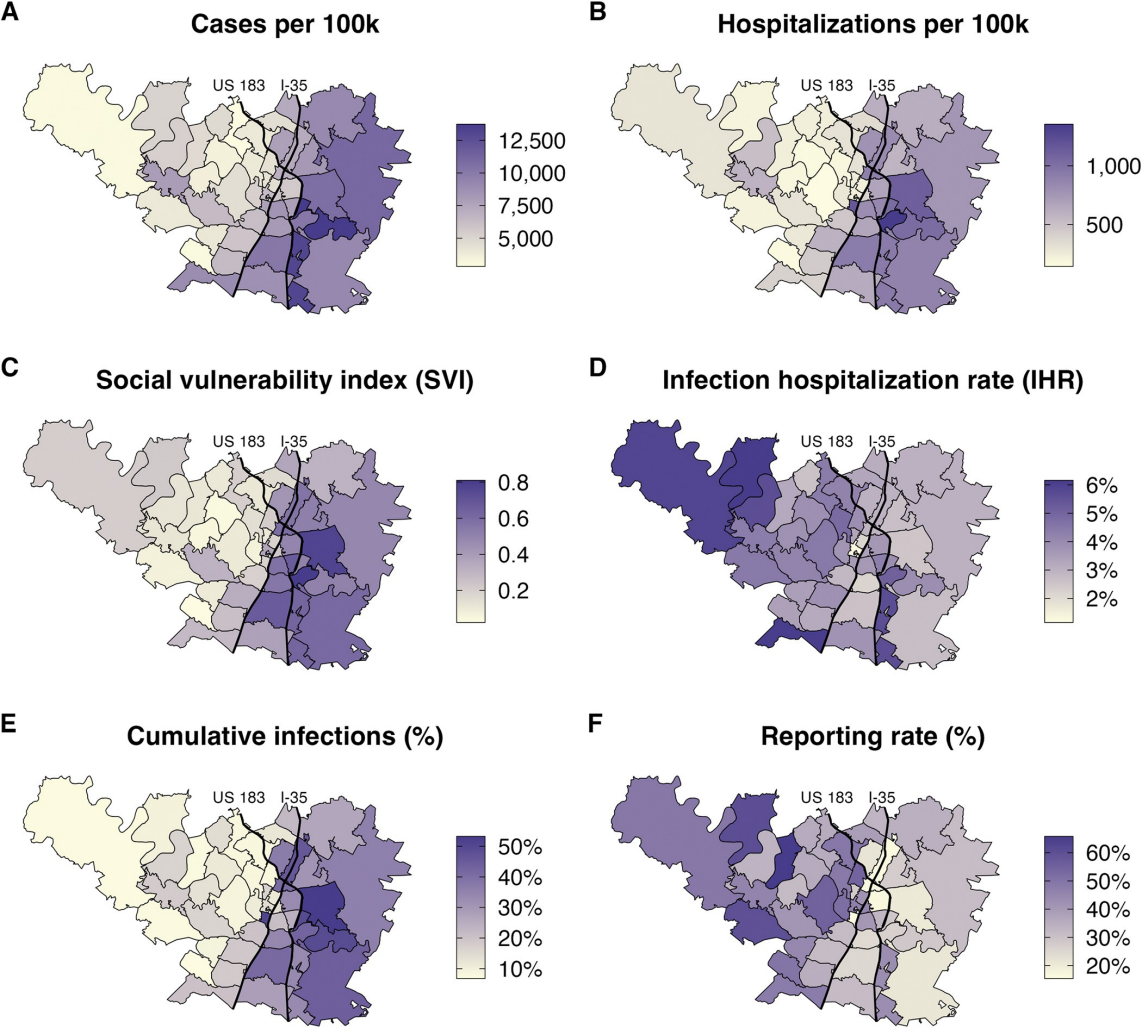
Reported and estimated COVID-19 burden by ZIP code for Travis County between March 1, 2020 and June 1, 2021. (A) Reported COVID-19 cases per 100,000. (B) Reported COVID-19 hospitalizations per 100,000. (C) Social Vulnerability Index [14] (D) Estimated infection hospitalization rate (IHR). (E) Estimated cumulative infections as of June 1, 2021. (F) Estimated percent of COVID-19 infections that were reported. Thin black curves indicate Interstate 35 and highway US 183. The ZIP code map was based on TIGER/Line shapefiles provided by the US Census Bureau [56] accessed through the tidycensus R package for the year 2019 [57].

Estimated COVID-19 burden varies significantly across ZIP codes within Travis County, with Interstate 35 roughly partitioning the county into high risk ZIP codes in the East and low risk ZIP codes in the West (Fig 3A and 3B). High COVID-19 risk visibly aligns with high social vulnerability, as measured by ZIP-code level SVI (Fig 3C). Our estimates for ZIP-code level infection hospitalization rates exhibit the opposite geographic trend (Fig 3D) from the absolute hospitalization rates (S6 Fig). We estimate that a ZIP code in east Austin (78724) had the highest infection rate of 53.7% (95% CrI: 42.7–67.1), while a Southwest Austin ZIP code (78739) had the lowest estimated infection rate of 4.8% (95% CrI: 2.6–8.5%) (Fig 3E) up to June 1, 2021. Downtown Austin (78701) had the lowest reporting rate of any ZIP code, with an estimated 15.2% (95% CrI: 11–20%) of infections reported, while a West Austin ZIP code (78732) had the highest reporting rate of 67% (95% CrI: 38–99%) (Fig 3F). Similar geographic patterns exist for each of the four age groups (S8–S10 Figs). Similar to previous work, we estimated the relationship between infections and reporting rate across ZIP codes using the function *r*= *a*∙*I*^−*b*^ and found a significant inverse relationship with *a* = 70.3 (95% CrI: 28–141) and *b* = 0.53 (95% CrI: 0.45–0.61) (S11 Fig) [55].

**Fig 4:**
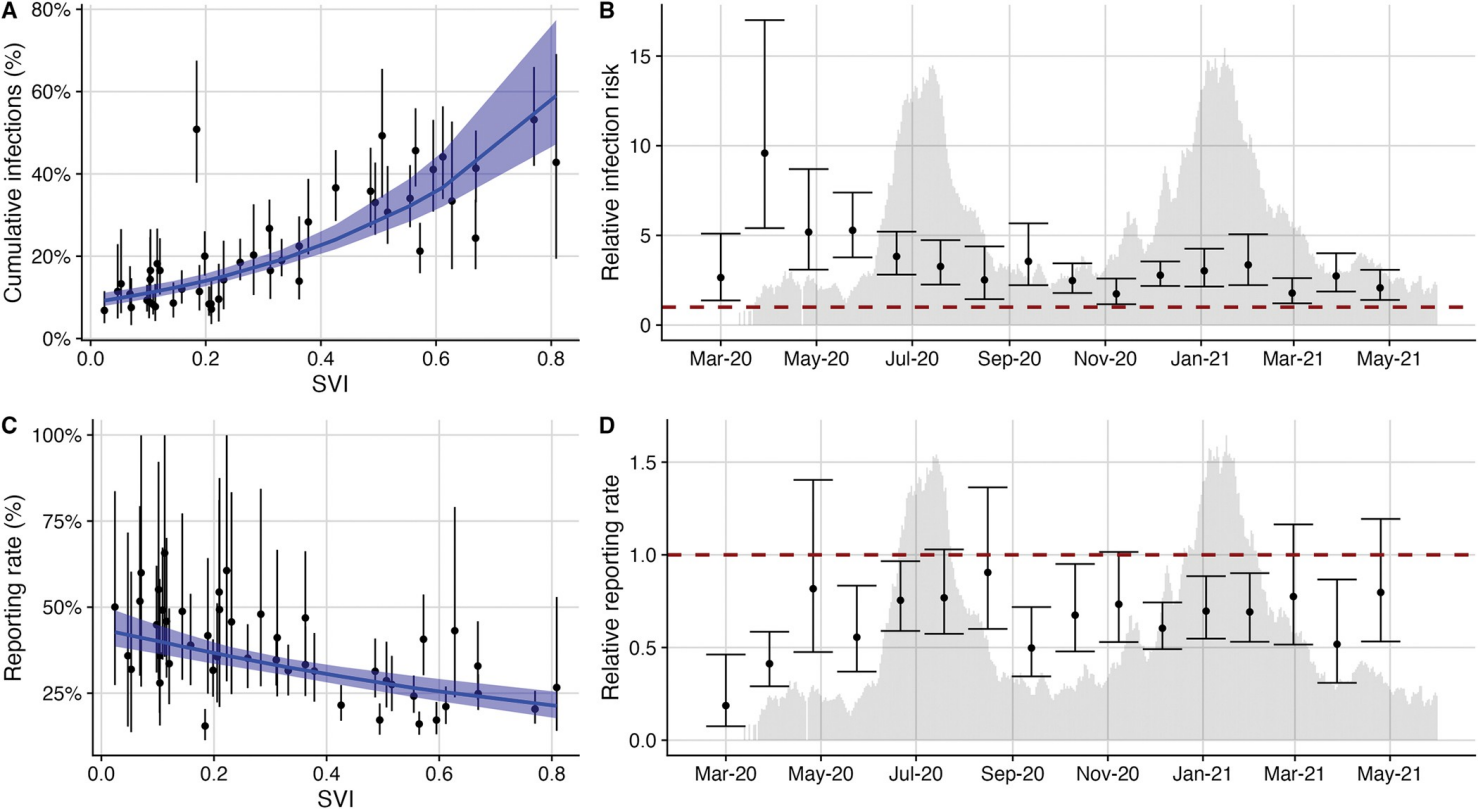
Infection and reporting rates correlate with social vulnerability in Travis County from March 1, 2020 to June 1, 2021. (A) Across the 46 ZIP codes, SVI is a significant predictor of estimated cumulative infections (p<0.001). The blue line and ribbon indicate the mean and 95% prediction interval from the fitted Poisson mixed-effects model. (B) Using the fitted model, we compare the expected infection rates among more and less vulnerable ZIP codes (specifically, ZIP codes at the 75th and 25th percentiles in the SVI distribution, respectively). The points indicate the expected ratio between these two values calculated using the estimated SVI regression coefficient from the 4-week time period; error bars indicate 95% CI’s. (C) Across the 46 ZIP codes, SVI is a significant predictor of estimated case reporting rates (p<0.001). The blue line and ribbon indicate the mean and 95% prediction interval from the fitted Poisson mixed-effects model. (D) Four week estimate for the inequality relationship between SVI and infection reporting rates across the 46 ZIP codes. Points and error bars show the mean and 95% CI for the relative reporting rate of individuals living in ZIP codes in the 75th SVI percentile compared with those living in the 25th SVI percentile. The red, horizontal dashed lines in B and D indicate if there were equitable infection risks or reporting rates across the 75th and 25th SVI percentile ZIP codes in the four week period. We overlay hospital admission time-series in B and D to showcase how inequality estimates compare with the progression of the local epidemic. For B and D we removed the ZIP codes reporting zero infections to stabilize the regression estimates.

The cumulative infection rates, case rates, and hospitalization rates are positively correlated with social vulnerability across Travis County’s 46 ZIP codes (Figs 4A and S12). Of the 15 individual components of SVI, we find that minority population rates, educational attainment rates, and household makeup are the strongest predictors of both infection rates (S13 Fig) and reporting rates (S14 Fig). We compare the relative risks for individuals living in a ZIP code at Travis County’s 25th (SVI = 0.12) and 75th (SVI = 0.5) percentile by SVI, where higher SVI indicates higher social vulnerability. Controlling for random ZIP code-level effects, we estimate that ZIP codes in the 75th SVI percentile experienced 2.5 (95% CrI: 2.0–3.0) times the infection rate of those in the 25th percentile. Similar trends are observed for each age group (S15 Fig and S1 Table). COVID-19 burden is often estimated directly from reported case or hospitalization data, without correcting for geographic biases in testing and underlying risk factors. For Travis county, we find that the subset of case data from APH provides a reasonable approximation but hospitalization data tends to inflate the estimated disparities (S1 Table). We aggregate the estimated number of infections occurring in each ZIP code into four-week periods from March 1, 2020 to June 1, 2021, and measure the relationship between SVI and the relative infection risk during this period. Significant disparity (i.e., a relative risk greater than one) persisted throughout the period and was highest during the first three months of the pandemic (Fig 4B). In April 2020, individuals living in the 75th SVI percentile ZIP code had an expected 9.6 (95% CrI: 5.4–17.0) times greater infection risk than those living in the 25th percentile SVI ZIP code. This ratio declined to 2.5 (95% CrI: 1.5–4.4) in August 2020 and hit a temporary minimum of 1.7 (95% CrI: 1.2–2.6) in November of 2020 before the large winter surge.

COVID-19 case reporting rates are negatively correlated with social vulnerability. We estimate that infections occurring in the 75th SVI percentile ZIP code were only 70% (95% CrI: 60%-82%) as likely to have been reported than those occurring in the 25th SVI percentile ZIP code. We further stratified by age group using a small sample of age-specific case data reported by the Austin Public Health community testing programs, which targeted vulnerable populations in East Austin (S16 Fig) [58]. We found that the negative correlation between SVI and case reporting rates held for all age groups except those over 65 years, perhaps because of Austin’s efforts to improve testing access for high risk individuals (S17 Fig and S1 Table). Throughout the study period, the estimated ratio in reporting rates between the 75th and 25th SVI percentile ZIP codes fluctuated, often dropping to levels significantly less than one (Fig 4D).

## Discussion

In the US, the first wave of the COVID-19 pandemic disproportionately harmed essential workers [5,6], residents of long-term care facilities [59], racial and ethnic minority populations [60], and socially vulnerable neighborhoods within cities [33,46,61,62]. Public health agencies and government officials have tried to address these disparities through targeted testing, vaccination, distribution of personal protective equipment, information campaigns, and paid sick leave [15–19]. Using a new method for inferring infection risks and reporting rates from COVID-19 hospital admissions data, we demonstrate that disparities persist on a granular scale within a large US city throughout the first year of the pandemic.

Our estimates for the spatial burden of COVID-19 in Austin, Texas suggest that children were less likely to be infected than adults under age 65 during the first major wave of transmission in the summer of 2020 but not during the subsequent winter wave. This is consistent with prior estimates [63–66] and may be attributable to early school closures, strict compliance with social distancing measures [67], or the emergence of variants that more efficiently infect children [68,69]. We also find that individuals over age 65 generally had the lowest risks of infection, despite suffering the highest per capita hospitalization rate, which may stem from heightened precautionary behavior and other protective measures such as COVID-19 screening in long-term care facilities [70]. Reporting rates were lowest in children and highest in older adults (Fig 2), which may stem from the positive correlation between age and symptom severity [63].

Our results suggest that, by June of 2021, the cumulative risk of infection in Travis county was about 23% lower than the average risk across the state of Texas (Figs 1 and S6 and S7). The divergence is consistent with a potential shift of COVID-19 burden from urban to rural regions in the United States [71], and may have stemmed from stricter COVID-19 mitigation policies or higher levels of public adherence in Travis County compared to the rest of Texas. In October 2020, the Texas governor issued Executive Order GA 32, which standardized COVID-19 policies across the state and limited local authority to enact restrictions [72]. In late 2020, Travis County enacted strict policies in violation of the statewide order which effectively mitigated a large winter surge [73,74]. In contrast, El Paso, Texas experienced a catastrophic surge in the fall of 2020 [75] and several other major Texas cities reported higher COVID infection, hospitalization and death rates than Austin during the winter of 2020–2021. While prior studies have shown positive COVID-19 epidemiological outcomes are associated with more stringent policies [76–78], the interaction between local and state policies and their impact on COVID-19 burden is not yet understood.

Historically marginalized populations in the “Eastern Crescent” of Austin were disproportionately harmed throughout the first year of the pandemic [79,80], mirroring disparities reported for Santiago, Chile and New York City [46,47,61]. After controlling for the higher prevalence of underlying risk factors in more vulnerable communities, we find that the ZIP codes ranking in the 75th percentile of social vulnerability had a more than twofold higher infection rate and a roughly 70% the case reporting rate than those ranking in the 25th percentile. Our estimates for inequity differ depending on the metric of analysis. For example, we estimate larger disparities between ZIP codes using raw hospitalization rates rather than infection rates, highlighting the importance of monitoring pandemic impacts across multiple indicators. In our analysis, higher hospitalization rate inequity may stem from the overlapping risks for infection and severe disease outcomes in vulnerable populations in Austin [60].

The estimated ratio in infection risk between more and less vulnerable regions decreased significantly during the first four months of the pandemic, perhaps because of local efforts to increase access to SARS-CoV-2 testing, isolation facilities, critical health information, and eventually vaccines [81]. The apparent decrease in disparity may also stem from higher infection rates in vulnerable populations leading to a more rapid buildup of immunity or relatively higher infection rates in less vulnerable areas during later time periods [82,83]. Exploring these hypotheses will be important to preventing disparities and protecting vulnerable populations during future infectious disease outbreaks. As of June of 2021, however, there remained a significant gap in COVID-19 risks and burden which informed targeted efforts by Austin Public Health to increase access to tests, vaccines, information and COVID-19 healthcare.

We developed this estimation method because of data availability. At the time, we were not able to obtain sub-city level seroprevalence data or reliable case counts, but had access to line-list hospitalization data indicating patient age and residential ZIP code. Given similar data, this approach can be applied to track longitudinal SARS-CoV-2 risks in cities and other geographic scales worldwide [84], as well as to estimate infection rates for other pathogens with high proportions of subclinical infections, such as influenza or Zika Virus (ZIKV) [85].

We note several assumptions of our analysis. First, the hospital admission data are limited by the accuracy of patient ZIP codes. Fewer than 1% of patients had unknown addresses. However, the missing data may correspond to vulnerable subgroups, such as people experiencing homelessness or undocumented residents, and thus obscure critical geographic or socioeconomic hotspots in our analysis. Second, we estimated each age- and ZIP-specific group independently rather than combining information across groups. This increases the uncertainty of our estimates but avoids the challenge of incorporating the changing contact and mobility patterns within the city throughout the pandemic [86–88]. Third, although we conducted analyses at a higher spatial resolution than most prior studies of COVID-19 burden, disparities in risk often occur at even more local scale [89]. Achieving COVID-19 health equity will require more granular surveillance and risk mitigation approaches.

Finally, we made the simplifying assumptions that the IHR was constant and that individuals could only be infected once. If reinfections were common during the analysis period, our model would underestimate the infection count. As such, we limited our analysis to the time period before the emergence of the Delta variant, after which reinfections and vaccine breakthrough infections were common [90–92]. Estimating local infection risks beyond June 2021 will require additional data and methods for accounting for reinfections and inferring the extent to which prior infection, prior vaccinations, and new variants modify the severity of infection.

We estimate that less than 25% of the Austin, Texas population was infected by SARS-CoV-2 prior to June 1, 2021 and that vulnerable communities in East Austin bore the brunt of the first two large waves of transmission. Our study introduces a framework for tracking infection and reporting rates on a granular scale using hospitalization data and provides evidence that the CDC’s social vulnerability index (SVI) is a strong predictor of risk that can inform targeted interventions.

## Materials and methods

We estimate the daily age-stratified numbers of infections for each of the 46 ZIP codes in Travis County, Texas from hospital linelist data provided by the three major local healthcare systems to Austin Public Health [93]. As described below, we first estimate age-specific infection hospitalization rates (IHRs) from state-wide COVID-19 hospitalization data and SARS-CoV-2 seroprevalence data and then use the IHR estimates to infer the number and timing of infections by age group and ZIP code. All code to recreate the analyses and figures can be accessed in the associated github repository (https://github.com/sjfox/austin-disparities).

### Estimating Texas statewide infection hospitalization rates (IHRs)

The infection hospitalization rate is defined as the proportion of infected individuals that are hospitalized. We used age-stratified COVID-19 hospitalization data [52] and SARS-CoV-2 antibody seroprevalence data [54] to estimate the age-specific infection hospitalization rate in Texas.

For each age group, we estimate the number of infections occurring between *t*_0_ (July 29, 2020) and *t*_1_ (May 27, 2021) according to a normal distribution as:

$$NI_{k,\mathrm{state}}∼\mathrm{normal}(\mathrm{mean}=I_{k,\mathrm{state}}(t_{1})-I_{k,\mathrm{state}}(t_{0}),\mathrm{var}=\sigma_{k,\mathrm{state}}^{2}(t_{0})+\sigma_{k,\mathrm{state}}^{2}(t_{1}))$$

Where *NI*_*k*,state_ corresponds to the estimated number of infections in the state in age group *k*, *I*_*k*,state_ corresponds to the mean CDC infection estimate at time, *t*, and

$$\sigma_{k,\mathrm{state}}^{2}(t)={\left(\frac{\max\{I_{k,\mathrm{state},97.5\%}(t)-I_{k,\mathrm{state}}(t),I_{k,\mathrm{state}}(t)-I_{k,\mathrm{state},2.5\%}(t)\}}{1.96}\right)}^{2}$$

Where I_k,state.2.5%_(t) and I_k,state.97.5%_(t) correspond to the lower and upper CDC infection confidence interval estimates respectively [53]. Texas seroprevalence samples were tested for SARS-CoV-2 anti-nucleocapsid antibodies during the time period of interest, so they only include individuals whose immunity derives from infection rather than vaccination [54].

We estimated statewide hospital admissions in the same time interval by accounting for the delay between infection and hospitalization. We fit a gamma distribution to the combined distribution derived from the time to symptom onset estimated in [94] and the time between symptom onset and hospital admission estimated in [95]. We chose the gamma distribution because it was able to match the non-normal shape of the combined distribution. We estimated the delay distribution as:

δ∼Γ(shape=2.99,rate=0.27)

We generated 1,000 samples for *δ* and generated a distribution of total hospital admissions for each age group as:

$$NH_{k,\mathrm{state},i}=\sum_{t=t_{0}+\delta_{i}}^{t_{1}+\delta_{i}}H_{k,\mathrm{state}}(t)$$

Where *NH*_*k*,state,*i*_ is a single sample of the hospital admission distribution and *H*_*k*,state_(*t*) is the raw hospital admission count for Texas at time, *t*. We aggregated hospital admission data, which are stratified into 0–17, 18–19, 20–29, 30–39, 40–49, 50–59, 60–69, 70–79, and 80+ year age groups, to match the stratification of the seroprevalence data (0–17, 18–49, 50–64, and 65+ years). For bins that do not align, we divided admissions evenly across years within a bin.

Finally, we estimated the infection hospitalization rate for each of the 1,000 samples as:

$$\mu_{k,\mathrm{state},i}=\frac{NH_{k,state,i}}{NI_{k,state,i}}$$

Where *μ*_*k*,state,*i*_ is a single sample of the infection hospitalization rate for age group *k* and *NI*_*k*,state,*i*_ is a single sample from the estimated normal distribution.

### ZIP- and age-specific infection hospitalization rates (IHRs)

Infection hospitalization rates depend on the underlying demographic makeup of a population [64]. To estimate age- and ZIP-specific IHRs from the statewide averages, we assumed that risk differences between ZIP codes could be captured by the proportion of the population estimated to be at high risk for severe COVID-19. We converted the statewide age-specific IHRs to ZIP-specific ones as:

$$\mu_{k,z}=\rho_{k,z}\cdot \mu_{k,\mathrm{state},\mathrm{hr}}+(1-\rho_{k,z})\cdot \mu_{k,\mathrm{state},\mathrm{lr}}$$

where *μ*_*k*,*z*_ is the infection hospitalization rate for age group, *k*, and ZIP code *z*, *μ*_*k*,state,hr_ and *μ*_*k*,state,lr_ are the statewide estimated age-specific IHRs for those at high and low risk to severe COVID-19 outcomes respectively, and *ρ*_*k*,*z*_ is the proportion of the population at high risk to severe COVID-19 outcomes in that age and zip code. The estimates of *ρ*_*k*,*z*_ represent the proportion of the population having at least one chronic condition linked to increased risk of severe COVID-19 disease such as cancer, obesity, diabetes, asthma, or HIV [5,96]. We assume a fixed hospitalization risk ratio between low and high risk individuals, μ_k,state,hr_ = η_k_·μ_k,state,lr_, where *η*_*k*_ is the age-specific hospitalization risk ratio estimated in [97]. For example, high risk individuals in the 20–24 and 75+ age groups are estimated to have 6.5 and 2.2 times the hospitalization risk respectively compared with low risk individuals in the same age group (S2 Table). We then estimate *μ*_*k*,state,lr_ and *μ*_*k*,state,hr_ as:

$$\mu_{k,\mathrm{state},\mathrm{lr}}=\frac{\mu_{k,\mathrm{state}}}{(\eta_{k}-1)\cdot \rho_{k,\mathrm{state}}+1}$$

$$\mu_{k,\mathrm{state},\mathrm{hr}}=\eta_{k}\cdot \mu_{k,\mathrm{state},\mathrm{lr}}$$

where *μ*_*k*,state_ is the estimated statewide age-specific IHR, and *ρ*_*k*,state_ is the statewide age-specific estimate for the proportion of the population at high risk for severe COVID-19 [5,96]. Confidence intervals for *μ*_*k*,z_ are derived by converting the lower and upper bound estimates for *μ*_*k*,state_ in the same fashion.

### Age- and ZIP code- specific infection estimates

We estimate the number of infections (*I*_*k*,*z*_) in a specific age group (*k*) and ZIP code (*z*) using the reported hospital admissions (*H*_*k*,*z*_) and the infection hospitalization rate (*m*_*k*,*z*_). We assume that infections are independent from one another and that every infected individual within an age group and ZIP code has the same chance of being hospitalized (*m*_*k*,*z*_). We describe their relationship with a binomial distribution as:

$$p(H_{k,z}|I_{k,z},m_{k,z})∼\mathrm{binom}(I_{k,z},m_{k,z})$$

We use a discrete uniform prior for *I*_*k*,*z*_ that ensures there are at least as many infections as hospital admissions (*H*_*k*,*z*_) and no more infections than the total population size (*N*_*k*,*z*_), and we assume an informative prior beta distribution for *m*_*k*,*z*_ as it is a flexible distribution that ensures the rate remains between zero and one:

$$p(m_{k,z})∼\mathrm{beta}(a_{k,z},b_{k,z})$$

$$p(I_{k,z})∼\mathrm{unif}(H_{k,z},N_{k,z})$$

We estimate the parameters for the informative beta prior distribution, *a*_*k*,*z*_ and *b*_*k*,*z*_, using the ZIP and age-specific IHR estimates estimated from seroprevalence data in the previous section. Specifically we use the equation:

$$a_{k,z}=\frac{b_{k,z}\cdot \mu_{k,z,\mathrm{mean}}}{1-\mu_{k,z,\mathrm{mean}}}$$

and identify the value of *b*_*k*,*z*_ that minimizes the difference between *μ*_*k*,*z*,2.5%_ and the 2.5th percentile of the resulting beta distribution, beta(*a*_*k*,*z*_, *b*_*k*,*z*_). In essence, we estimate the shape parameters of a beta distribution that match the mean and lower bound estimate of the IHR.

Our posterior distribution for *I*_*k*,*z*_ and *m*_*k*,*z*_ can then be defined as:

$$p(I_{k,z},m_{k,z}|H_{k,z})\propto p(H_{k,z}|m_{k,z},I_{k,z})\cdot p(I_{k,z})\cdot p(m_{k,z})$$

We used Markov chain Monte Carlo (MCMC) sampling of the posterior distribution using the rjags package in the R programming language [98,99]. Specifically we sample 1,000 draws from the posterior distribution across four chains thinning every two samples and with a 200 sample burn-in period. Throughout the paper we summarize the posterior distributions using their mean and 95% credible intervals.

### Estimating the timing of infections

We created a distribution of the infection timing using the hospital admission timing and the previously estimated delay distribution between infection and hospital admission, δ~Γ(shape =2.99,rate =0.27).

We draw 1,000 infection time-series samples using the 1,000 posterior infection samples for each age- and ZIP-group. For each sample and for infection i∈{1,…,I_k,z_} we:

1. Draw a single hospital admission date, *d*_*H*,*i*_, randomly from the dates of all hospital admissions recorded in the age- and ZIP-group
2. Draw a single infection to hospital admission delay, δ_*i*_, from the delay distribution
3. Assign the date of the infection, *d*_*I*,*i*_, as *d*_*I*,*i*_ = *d*_*H*,*i*_−*δ*_*i*_

Each of the 1,000 estimated d_I_ vectors capture a single infection time-series. For age and ZIP code groups that had estimated infections but zero reported hospitalizations, we drew the hospital admission date, *d*_*H*,*i*_, from the dates of all hospital admissions within the ZIP code. We present an example outlining the infection estimation procedure in the supplement (S18 Fig).

### Reporting rate estimates

We assumed that every infection, *I*_*j*_, had the same chance of being reported, *r*_*j*_, and that reported cases, *C*_*j*_, were independent from one another, so that reported cases were distributed binomially as:

$$p(C_{j})∼\mathrm{binom}(I_{j},r_{j})$$

Where *j* describes the specific subgroup of interest (age group, *k*, and/or ZIP code, *z*). Assuming a uniform conjugate beta prior distribution on the reporting rate, the posterior for *r*_*j*_ can be calculated as:

$$p(r_{j}|I_{j},C_{j})∼\mathrm{beta}(1+C_{j},1+I_{j}-C_{j})$$

For subgroups where *I*_*j*_<*C*_*j*_, we increase *I*_*j*_ so that *I*_*j*_ = *C*_*j*_. We estimated overall and subgroup reporting rates for the full study period through June 1, 2021 using cumulative age-specific case counts for Travis County [100] as well as ZIP code specific counts provided by Austin Public Health (APH) [101]. Separately, we estimated the age- and ZIP-specific reporting rates from a subset of testing data provided to us directly from Austin Public Health, which included 60% of reported cases in Travis County during the time period.

### Social Vulnerability Index (SVI) as a predictor

The CDC’s Social Vulnerability Index (SVI) is an indicator that estimates a community’s ability to withstand a hazardous emergency event such as a hurricane or disease outbreak [14]. SVI values are based on 15 different American Community Survey (ACS) variables and are given at the level of census tract as percentile ranks (range 0.0–1.0) within each state based on the 2014–2018 5-year ACS. For example, an SVI of 0.6 indicates that a census tract is more vulnerable than 60% of other census tracts in the state. We aggregated SVI to ZIP codes using weighted averages based on the percent of residential addresses in a ZIP code that fall in each census tract [102,103].

We estimated the impact of SVI on infection and reporting rates using a mixed effect poisson regression model using the lme4 R package [104]. For estimating the impact of SVI on infection rates the model can be described as:

$$I_{z,i}∼\mathrm{Pois}\left(\exp(\beta_{I,1}\cdot SVI_{z}+\beta_{I,0}+\pi_{I,z})\right)$$

Where *I*_*Z*,*i*_ is the *i*th infection estimate sample estimated for ZIP code, *z*, *SVI*_*z*_ is the ZIP codes’ SVI, *π*_*I*,*z*_ is the ZIP code level random effect, *β*_*I*,1_ is the fixed effect of SVI on infections, and *β*_*I*,0_ is an intercept term. We use the ZIP code population as an offset in the model to standardize infection rates. For estimating the impact of SVI on reporting rates the model can be described as:

$$C_{z}∼\mathrm{Pois}\left(\exp(\beta_{C,1}\cdot SVI_{z}+\beta_{C,0}+\pi_{C,z})\right)$$

where *C*_*z*_ is the reported case count for ZIP code, *z*, *π*_*C*,*z*_ is the ZIP code level random effect, *β*_*C*,1_ is the fixed effect of SVI on cases, and *β*_*C*,0_ is an intercept. We use the 1,000 ZIP code infection estimate samples as an offset in the model to standardize reporting rates. For the age and ZIP code-stratified analysis we also include an interaction term between age and SVI, so the equations become:

$$I_{k,z,i}∼\mathrm{Pois}\left(\exp(\beta_{I^{\prime },k,1}\cdot SVI_{z}+\beta_{I^{\prime },0}+\pi_{I^{\prime },z})\right)$$

$$C_{k,z}∼\mathrm{Pois}\left(\exp(\beta_{C^{\prime },k,1}\cdot SVI_{z}+\beta_{C^{\prime },0}+\pi_{C^{\prime },z})\right)$$

where *I*_*k*,*z*,*i*_ is the *i*th infection estimate sample in age group, *k* for ZIP code, *z*, *C*_*k*,*z*_ is the reported cases in age group, *k* for ZIP code, *z*, *β*_*I*′,*k*,1_ and *β*_*C*′,*k*,1_ are the SVI regression coefficient for age group, *k* for the infection and case interaction terms respectively, *β*_*I*′,0_ and *β*_*C*′,0_ are the intercepts, and *π*_*I*′,*z*_ and *π*_*C*′,*z*_ are the ZIP code level random effects. For the infection and reporting rate models we use the age- and ZIP- population and infection estimates as offsets respectively.

The SVI regression coefficients (*β*_*I*,1_, *β*_*C*,1_, *β*_*I*′,*k*,1_, and *β*_*C*′,*k*,1_) can be interpreted as inequality metrics, quantifying the relative infection and reporting risks as a function of SVI. Because there are no ZIP codes with a value of 0 or 1 for SVI in our sample, we report the relative infection and reporting rates between ZIP codes in the 25th and 75th percentile in Travis County throughout the manuscript.

## Supporting information

S1 Fig Daily COVID-19 burden estimates for Travis County, Texas from March 1, 2020 until June 1, 2021. Daily new reported case (A) and mortality (B) counts as reported by the New York Times for Travis County, Texas [100].
(TIFF)

S2 Fig Comparison of age-dependent estimates for infection-hospitalization rates. Age-stratified estimates of the risk of severe COVID-19 (defined as risk for hospitalization) from China [51], France [45].
(TIFF)

S3 Fig Weekly estimated relative infection rates from March 1, 2020 until June 1, 2021 across age groups. Points and error bars indicate the median and 95% confidence interval for the weekly infection rate with the size of the population. Values of 1 (horizontal dashed line) indicate that the fraction of the infections occurring that week equals the population fraction for the specific age group, while values below or above one indicate the age group faced disproportionately low or high infection risk respectively during that week. Only the 65+ age group consistently experienced disproportionately low infection rates compared with their population size over the whole pandemic.
(TIFF)

S4 Fig Reported 7-day average of case counts by age group from April 22, 2020 until May 28, 2021. Daily reported cases counts for each age group provided by Austin Public Health [101].
(TIFF)

S5 Fig Reported hospital admissions by age group from March 1, 2020 until June 1, 2021. Age-specific admission data provided by Austin Public Health.
(TIFF)

S6 Fig COVID-19 estimated cumulative infections for Travis County (Austin, TX) and the state of Texas from March 1, 2020 to June 1, 2021. Estimated cumulative infections in Travis County with 95% credible intervals (black line and gray ribbon) compared to Texas statewide seroprevalence-based estimates (red points and error bars) for each of the four age groups [49].
(TIFF)

S7 Fig Relative COVID-19 infection risk by age group in Travis County as compared to the state of Texas from March 1, 2020 to June 1, 2021. For each age group, we compare the mean model-estimated infection rates for Travis County with the mean statewide seroprevalence estimates in Texas to estimate the mean relative infection risks between the two (points and smoothed lines). Values above the horizontal dashed line indicate that Travis County residents faced higher infection risks than residents of Texas, while values below the line indicate higher statewide infection risks. As of June 1, 2021, infection rates were 45% (95% CrI: 20–61%), 19.5% (95% CrI: 0.1–33%), 22.7 (0.1–40%), and 29.8% (95% CrI: 2–48%) lower for individuals 0–17, 18–49, 50–64, and 65+ respectively in Travis compared with Texas as whole.
(TIFF)

S8 Fig Estimated ZIP code and age-specific IHR for each ZIP code in Travis County. Infection hospitalization rates derived from Texas-specific estimates (Table 1) using population risk estimation methodology for each age group as detailed in [5,96,105].
(TIFF)

S9 Fig Cumulative infection estimates for each ZIP code and age group in Travis County using hospitalization data up to June 1, 2021. (TIFF)

S10 Fig Cumulative estimated reporting rate for each ZIP code and age group in Travis County using reported case data up to June 1, 2021. Testing data used for reporting rates are only a subset of all tests performed, as age and ZIP code stratified data were only available for Austin Public Health administered tests.
(TIFF)

S11 Fig Relationship between the infections per 100,000 and the reporting rate for ZIP codes in Travis County (Austin, TX), between March 1, 2020 and June 1, 2021. Points and error bars indicate the mean and 95% confidence intervals for each ZIP code. The dashed line indicates the mean estimated relationship across 1,000 posterior samples, with a = 70.4 (95% CrI: 28–141) and b = 0.533 (95% CrI: 0.45–0.61).
(TIFF)

S12 Fig Reported case and hospitalization counts correlate with social vulnerability in Travis County from March 1, 2020 to June 1, 2021. (A) Across the 46 ZIP codes, SVI is a significant predictor of reported case counts (p<0.001). The blue line and ribbon indicate the mean and 95% prediction interval from the fitted Poisson mixed-effects model. (B) Across the 46 ZIP codes, SVI is a significant predictor of reported hospitalization counts (p<0.001). The blue line and ribbon indicate the mean and 95% prediction interval from the fitted Poisson mixed-effects model.
(TIFF)

S13 Fig Comparison between the estimated SARS-CoV-2 infection rates in each Travis County ZIP code from March 1, 2020 to June 1, 2021 with the 15 individual components of the Social Vulnerability Index (SVI). Points and error bars indicate the mean and 95% credible intervals for estimated infection rates in a ZIP code.
(TIFF)

S14 Fig Comparison between the estimated SARS-CoV-2 reporting rates in each Travis County ZIP code from March 1, 2020 to June 1, 2021 with the 15 individual components of the Social Vulnerability Index (SVI). Points and error bars indicate the mean and 95% credible intervals for estimated reporting rates in a ZIP code.
(TIFF)

S15 Fig Estimated infection rates correlate with social vulnerability in Travis County from March 1, 2020 to June 1, 2021 across all age groups. Across the 46 ZIP codes, SVI has a positive relationship with cumulative infection rates as a proportion of the population for every age group (S1 Table). Estimated age-specific SVI relationships from the poisson mixed effects regression model are shown in the blue line (mean) and blue ribbon (95% confidence interval).
(TIFF)

S16 Fig Observed biases in the subset of reported case data stratified by age and ZIP code. (A) Fraction of all reported cases included in the subset of age- and ZIP-code stratified data collected through Austin Public Health’s community testing programs by ZIP code. Overall, the data set covers 60% of all reported cases, but the data set, which does not include all cases identified by private testing sites, has high levels of coverage in the vulnerable ZIP codes of East Austin. (B) Reported case coverage from the dataset correlates positively with SVI. Blue line indicates the mean of a fitted linear regression model.
(TIFF)

S17 Fig Estimated reporting rates correlate with social vulnerability in Travis County from March 1, 2020 to June 1, 2021. Across the 46 ZIP codes, SVI has a flat or slightly negative relationship with cumulative infection reporting rates for every age group except for those aged 65+ (S1 Table). Estimated age-specific SVI relationships from the poisson mixed effects regression model are shown in the blue line (mean) and blue ribbon (95% confidence interval).
(TIFF)

S18 Fig Infection estimation methodology for a hypothetical region with 150 hospital admissions and a mean infection hospitalization rate of 0.2. (A) Prior distribution of the infection hospitalization rate for the example region specified by **α = 25** and **β = 100**. (B) Hospital admission counts by day in the example region. (C) Estimated cumulative infection distribution for the region based on the hospital admission count and IHR distribution. IHR distribution is made up of 1,000 draws from the posterior distribution. (D) Cumulative estimated infections over time for each of the 1,000 posterior infection draws. Timing is based on the hospital admission timing and the delay distribution between infection and hospitalization.
(TIFF)

S1 Table Comparison of age-stratified risk ratio between more vulnerable (75th SVI percentile) and less vulnerable (25th SVI percentile) ZIP codes for Travis county, from March 1, 2020 to June 1, 2021. Estimates based on reported COVID-19 hospitalizations are consistently higher than those based on model-derived estimates of ZIP-code level infection rates and observed COVID-19 case rates.
(XLSX)

S2 Table Relative hospitalization rates for high risk individuals compared with low risk individuals from [97]. (XLSX)
