# Supplementary figures and images for: Disproportionate impacts of COVID-19 in a large US city
Source: PLoS Comput Biol. 2023 Jun 1;19(6):e1011149. doi: 10.1371/journal.pcbi.1011149 (PMC10234557; doi:10.1371/journal.pcbi.1011149)

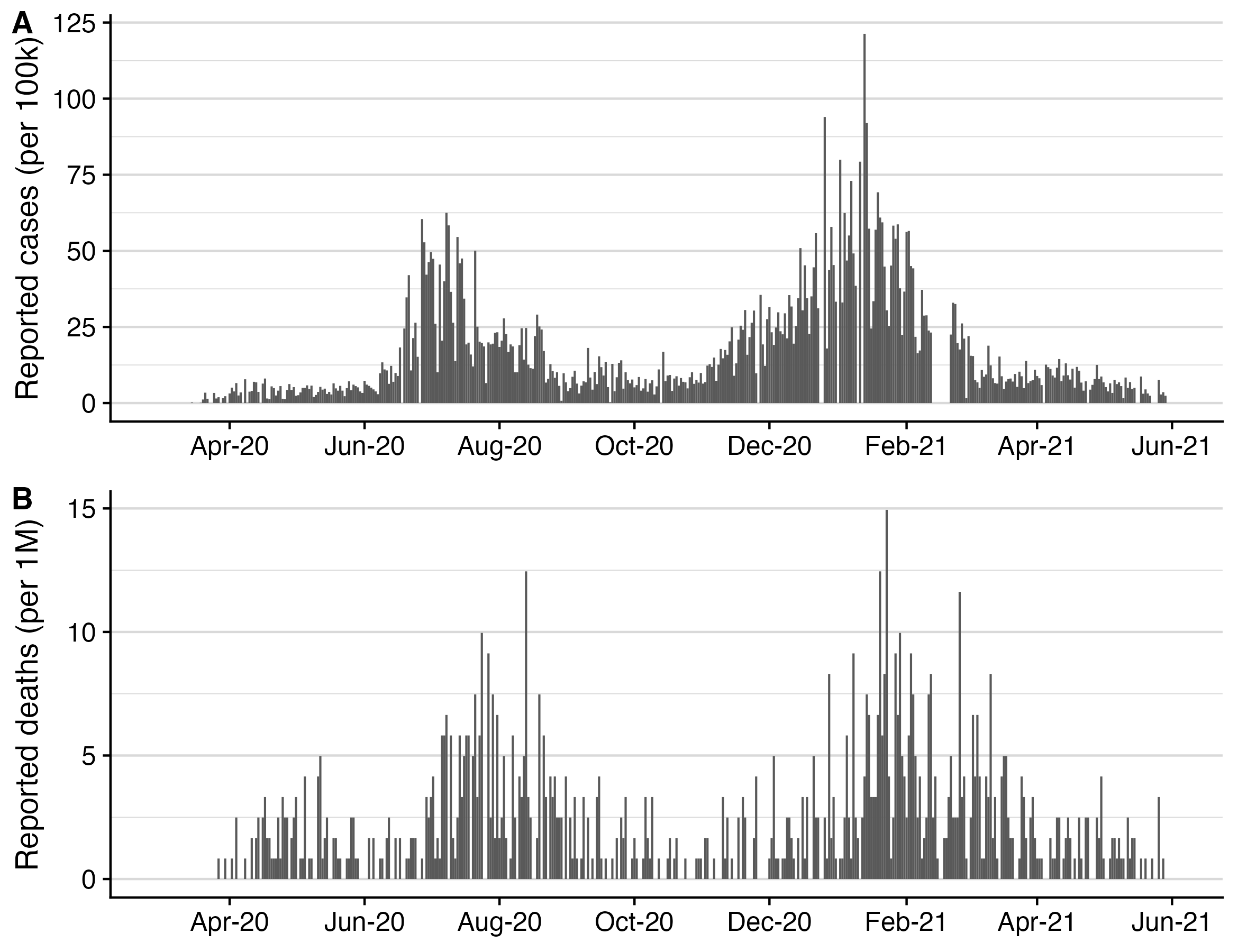

Supplement: S1 Fig — Daily new reported case (A) and mortality (B) counts as reported by the New York Times for Travis County, Texas [100]. (TIFF) [file pcbi.1011149.s001.tiff]

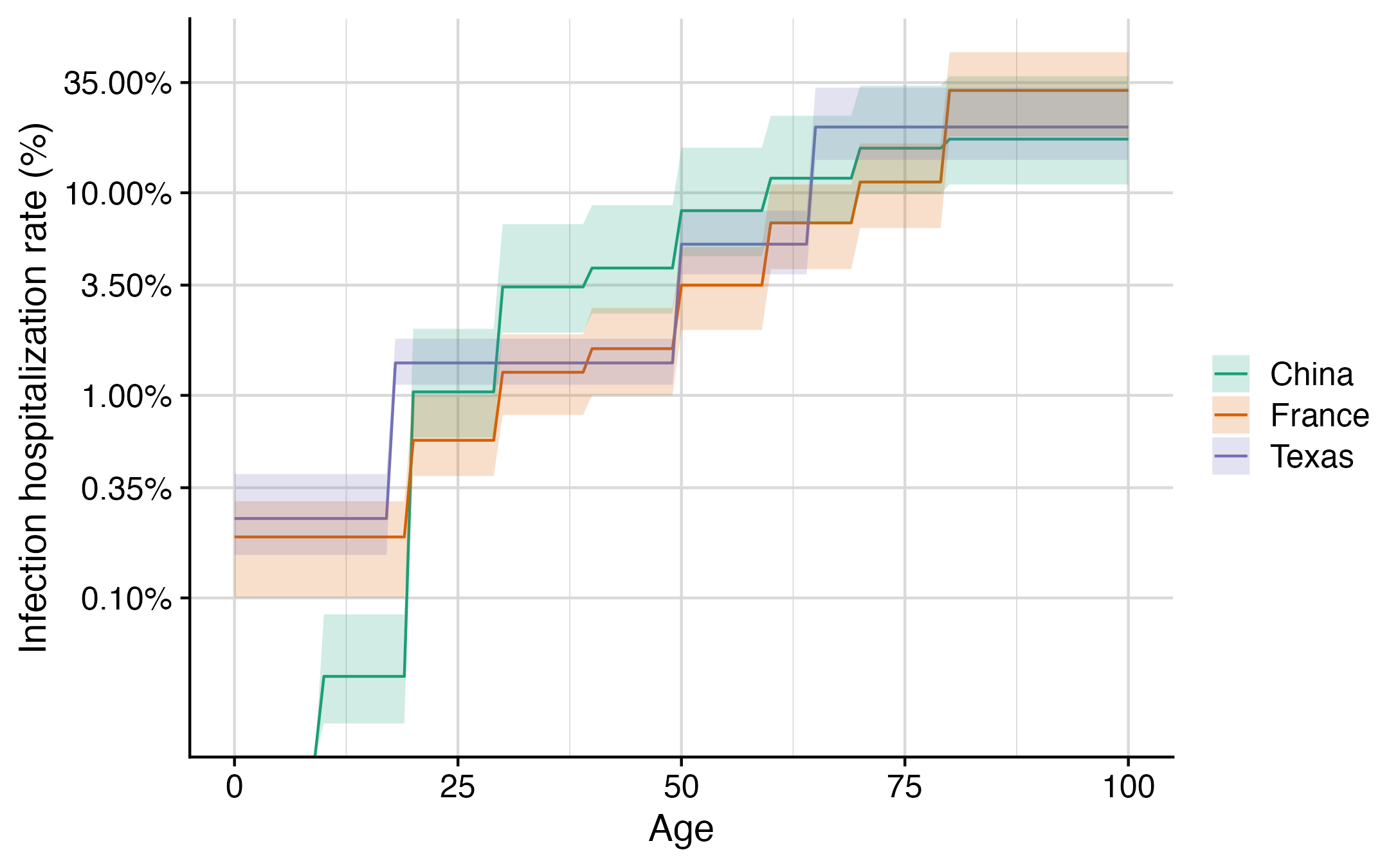

Supplement: S2 Fig — Age-stratified estimates of the risk of severe COVID-19 (defined as risk for hospitalization) from China [51], France [45]. (TIFF) [file pcbi.1011149.s002.tiff]

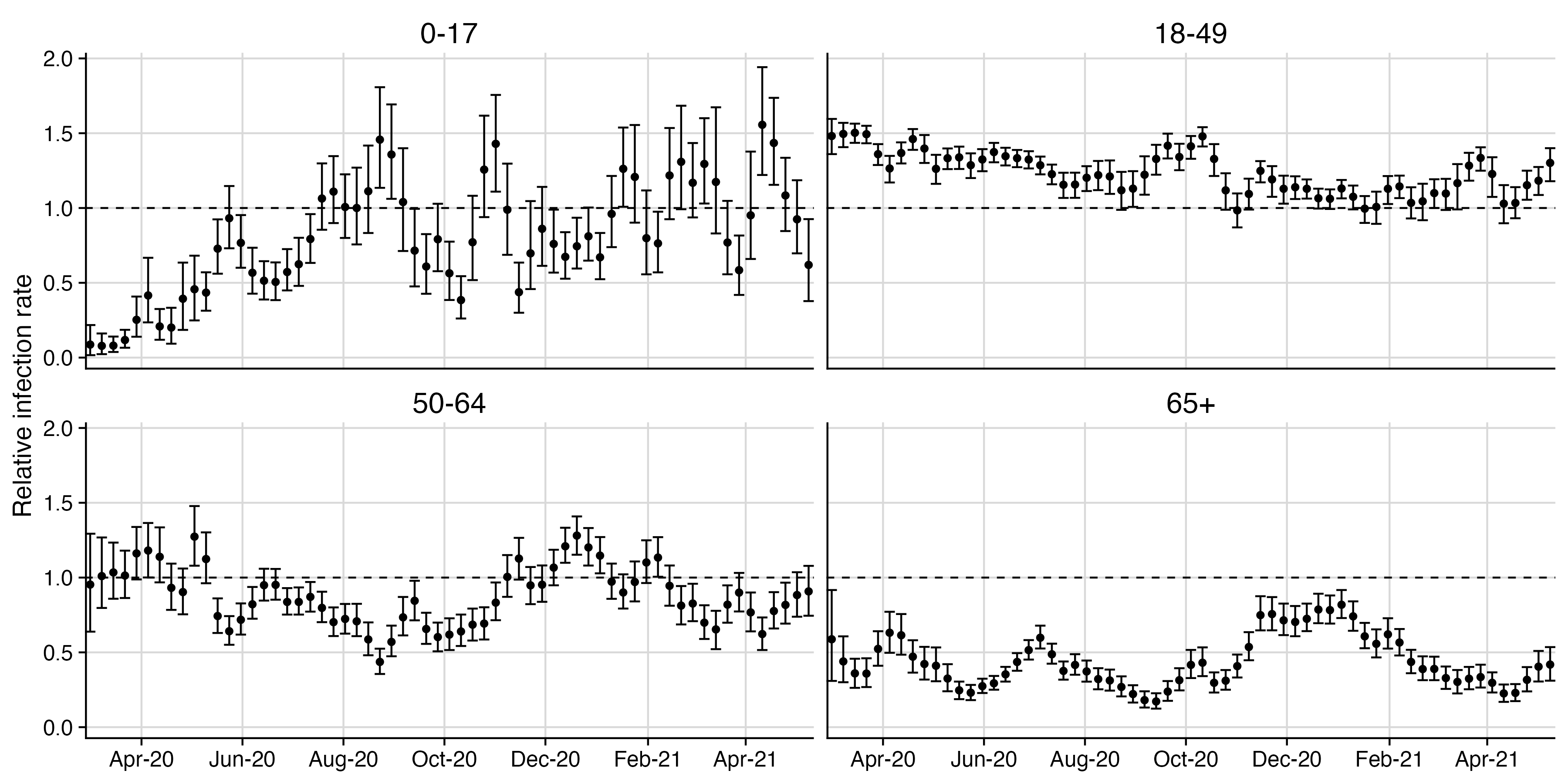

Supplement: S3 Fig — Points and error bars indicate the median and 95% confidence interval for the weekly infection rate with the size of the population. Values of 1 (horizontal dashed line) indicate that the fraction of the infections occurring that week equals the population fraction for the specific age group, while values below or above one indicate the age group faced disproportionately low or high infection risk respectively during that week. Only the 65+ age group consistently experienced disproportionately low infection rates compared with their population size over the whole pandemic. (TIFF) [file pcbi.1011149.s003.tiff]

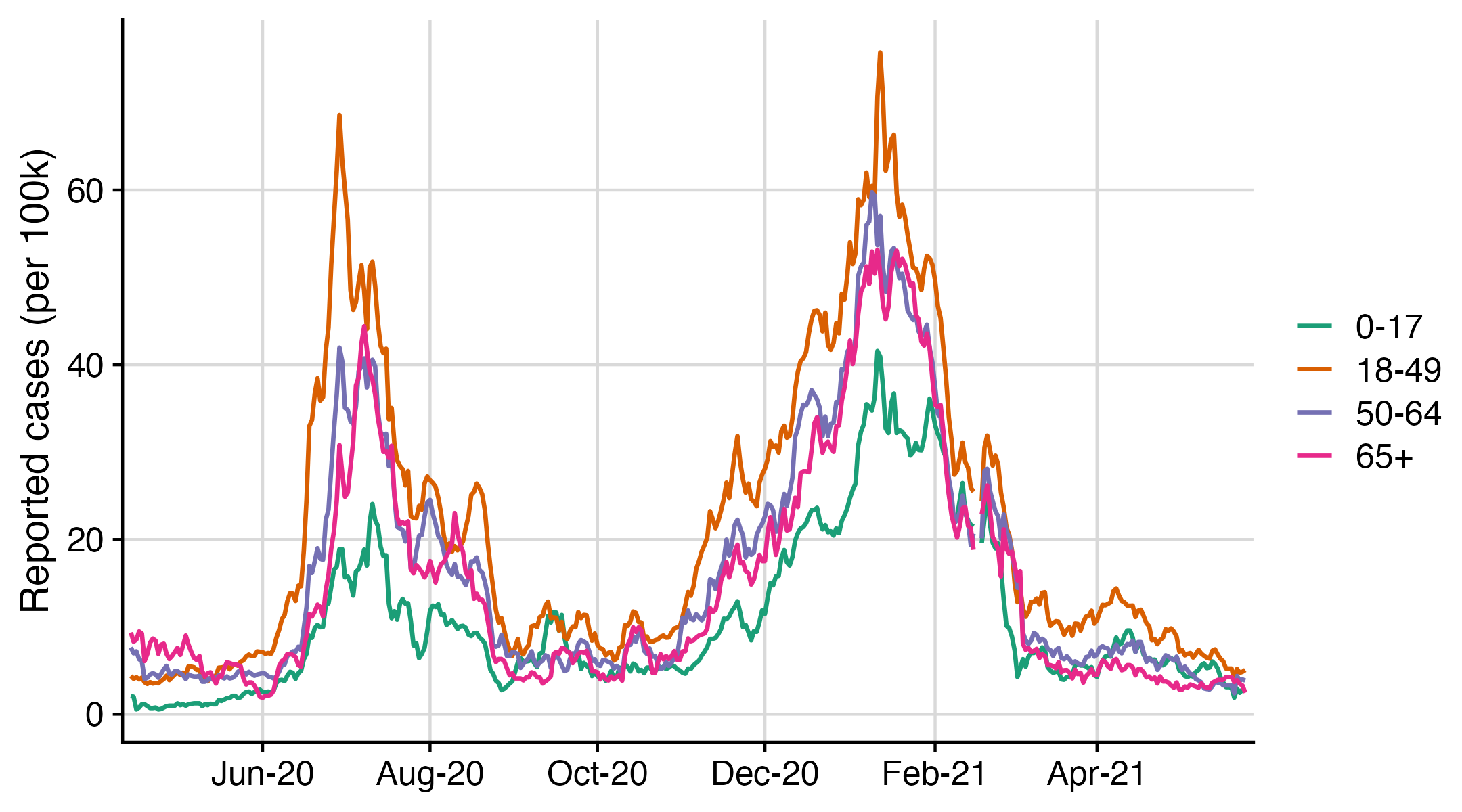

Supplement: S4 Fig — Daily reported cases counts for each age group provided by Austin Public Health [101]. (TIFF) [file pcbi.1011149.s004.tiff]

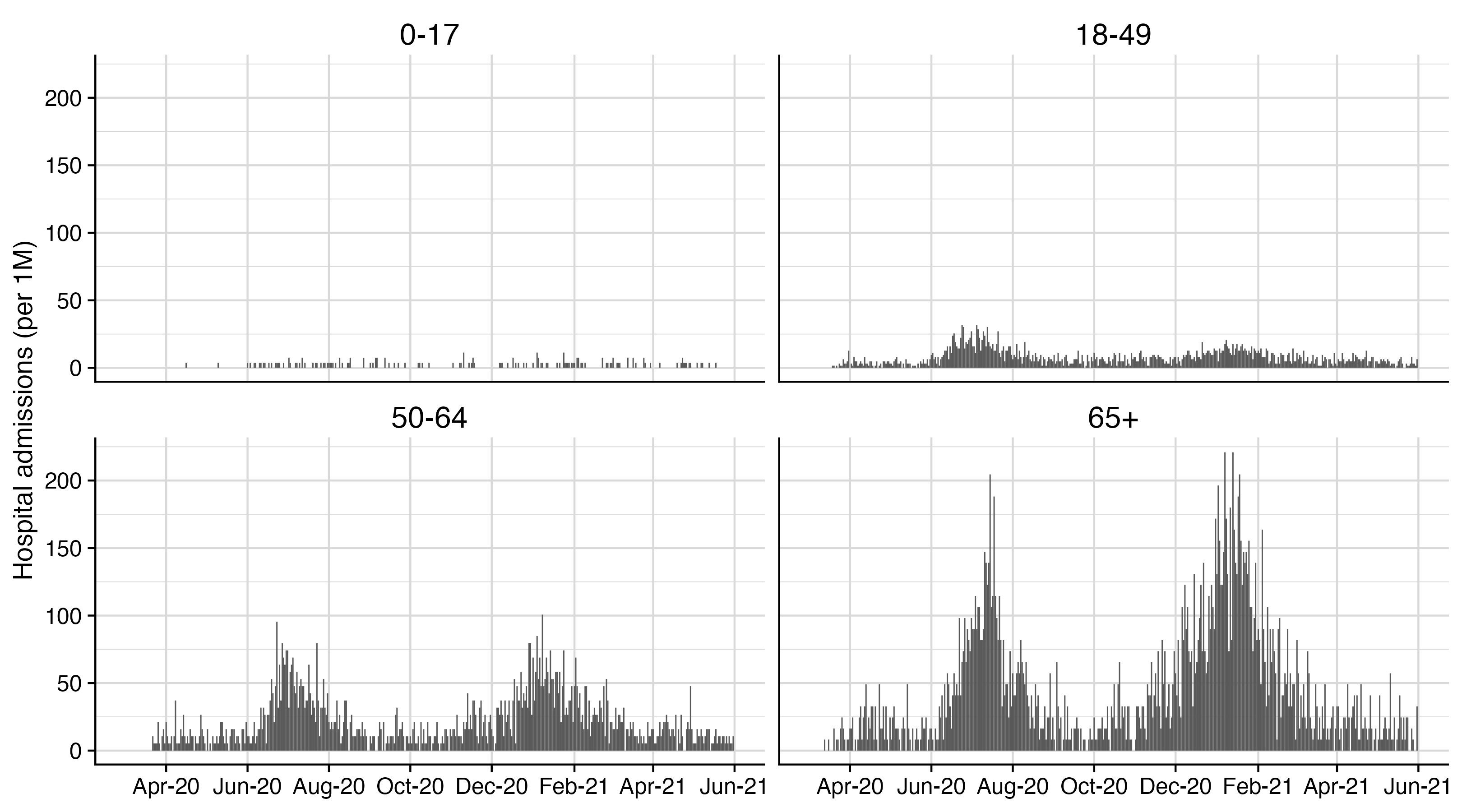

Supplement: S5 Fig — Age-specific admission data provided by Austin Public Health. (TIFF) [file pcbi.1011149.s005.tiff]

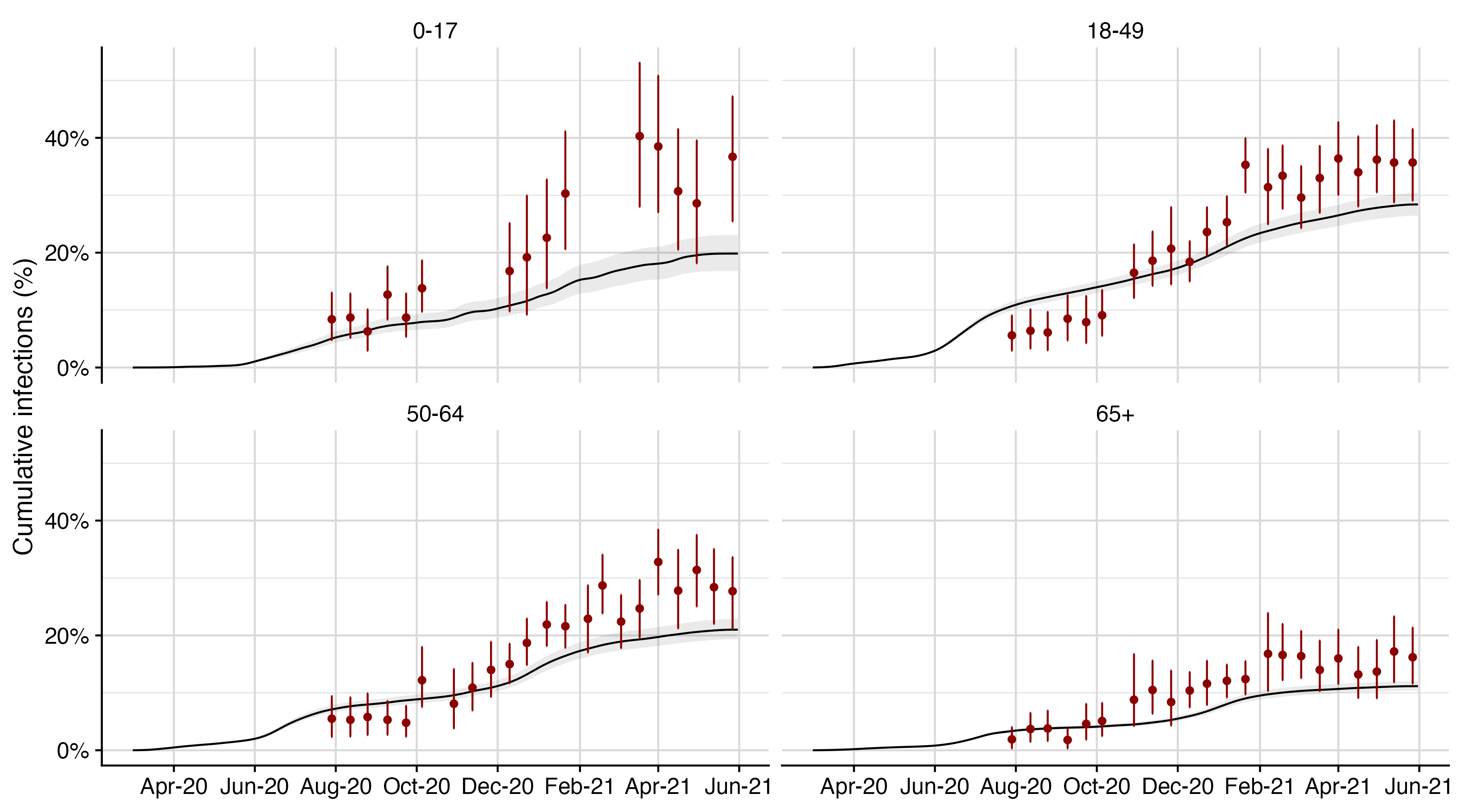

Supplement: S6 Fig — Estimated cumulative infections in Travis County with 95% credible intervals (black line and gray ribbon) compared to Texas statewide seroprevalence-based estimates (red points and error bars) for each of the four age groups [49]. (TIFF) [file pcbi.1011149.s006.tiff]

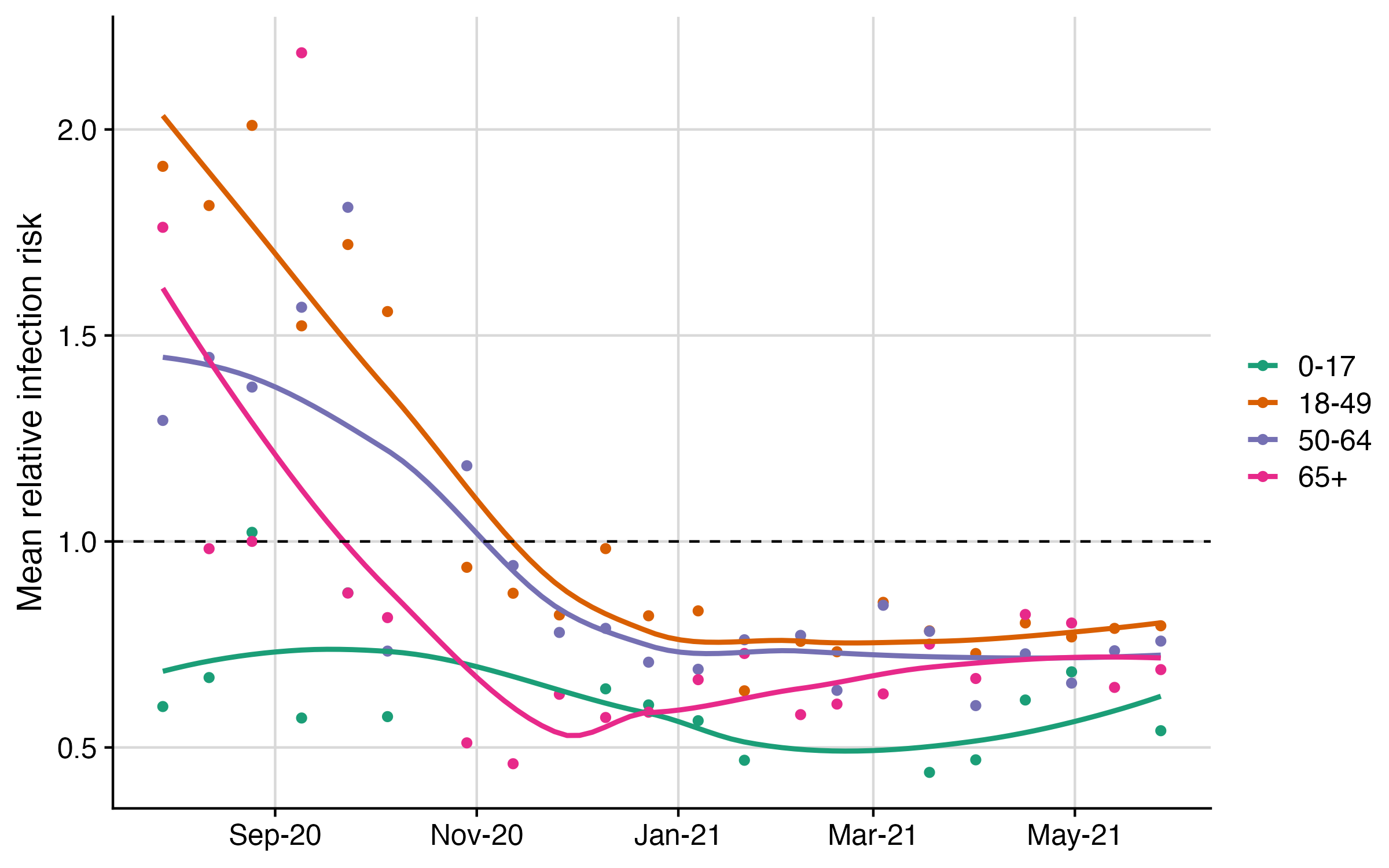

Supplement: S7 Fig — For each age group, we compare the mean model-estimated infection rates for Travis County with the mean statewide seroprevalence estimates in Texas to estimate the mean relative infection risks between the two (points and smoothed lines). Values above the horizontal dashed line indicate that Travis County residents faced higher infection risks than residents of Texas, while values below the line indicate higher statewide infection risks. As of June 1, 2021, infection rates were 45% (95% CrI: 20–61%), 19.5% (95% CrI: 0.1–33%), 22.7 (0.1–40%), and 29.8% (95% CrI: 2–48%) lower for individuals 0–17, 18–49, 50–64, and 65+ respectively in Travis compared with Texas as whole. (TIFF) [file pcbi.1011149.s007.tiff]

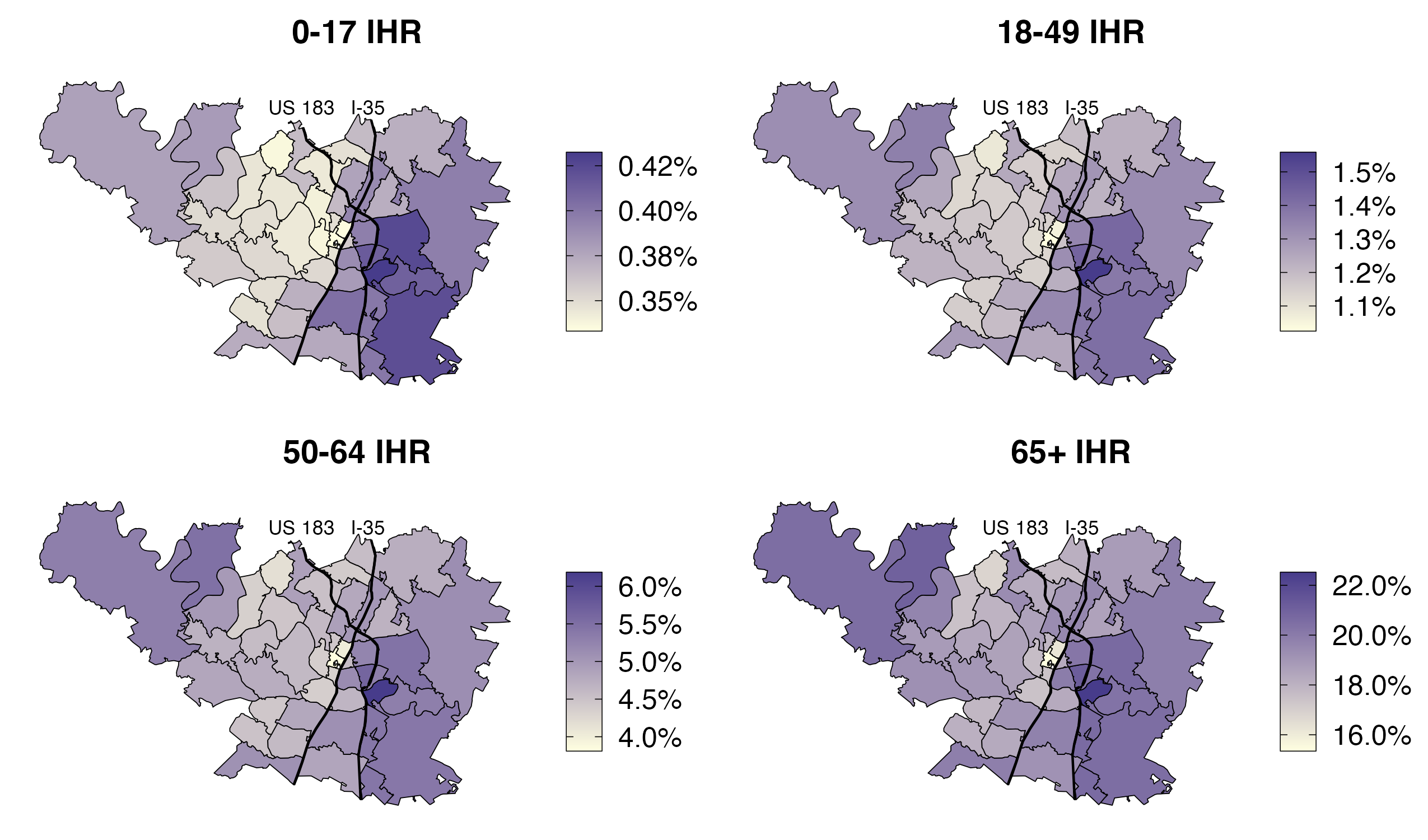

Supplement: S8 Fig — Infection hospitalization rates derived from Texas-specific estimates (Table 1) using population risk estimation methodology for each age group as detailed in [5,96,105]. (TIFF) [file pcbi.1011149.s008.tiff]

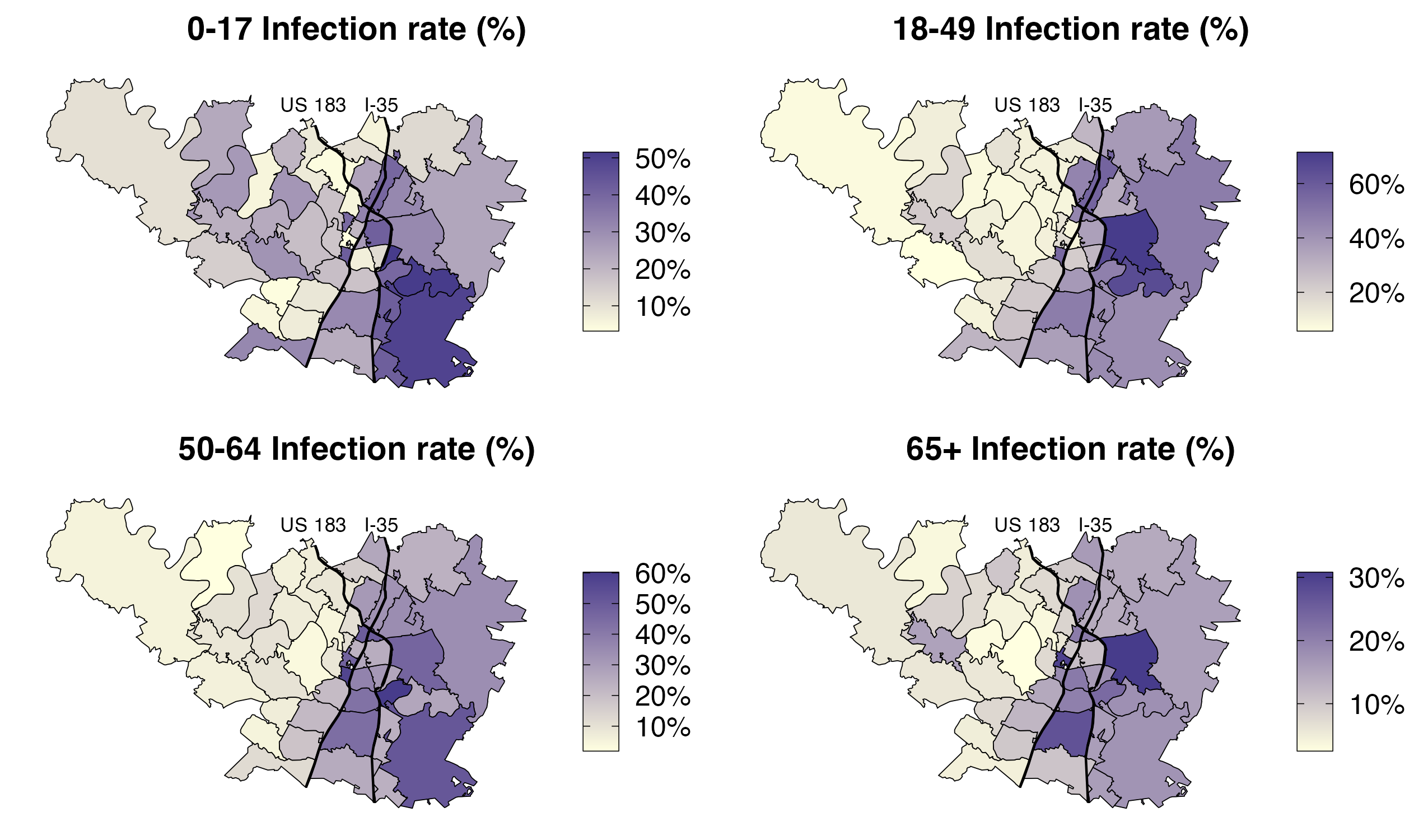

Supplement: S9 Fig — (TIFF) [file pcbi.1011149.s009.tiff]

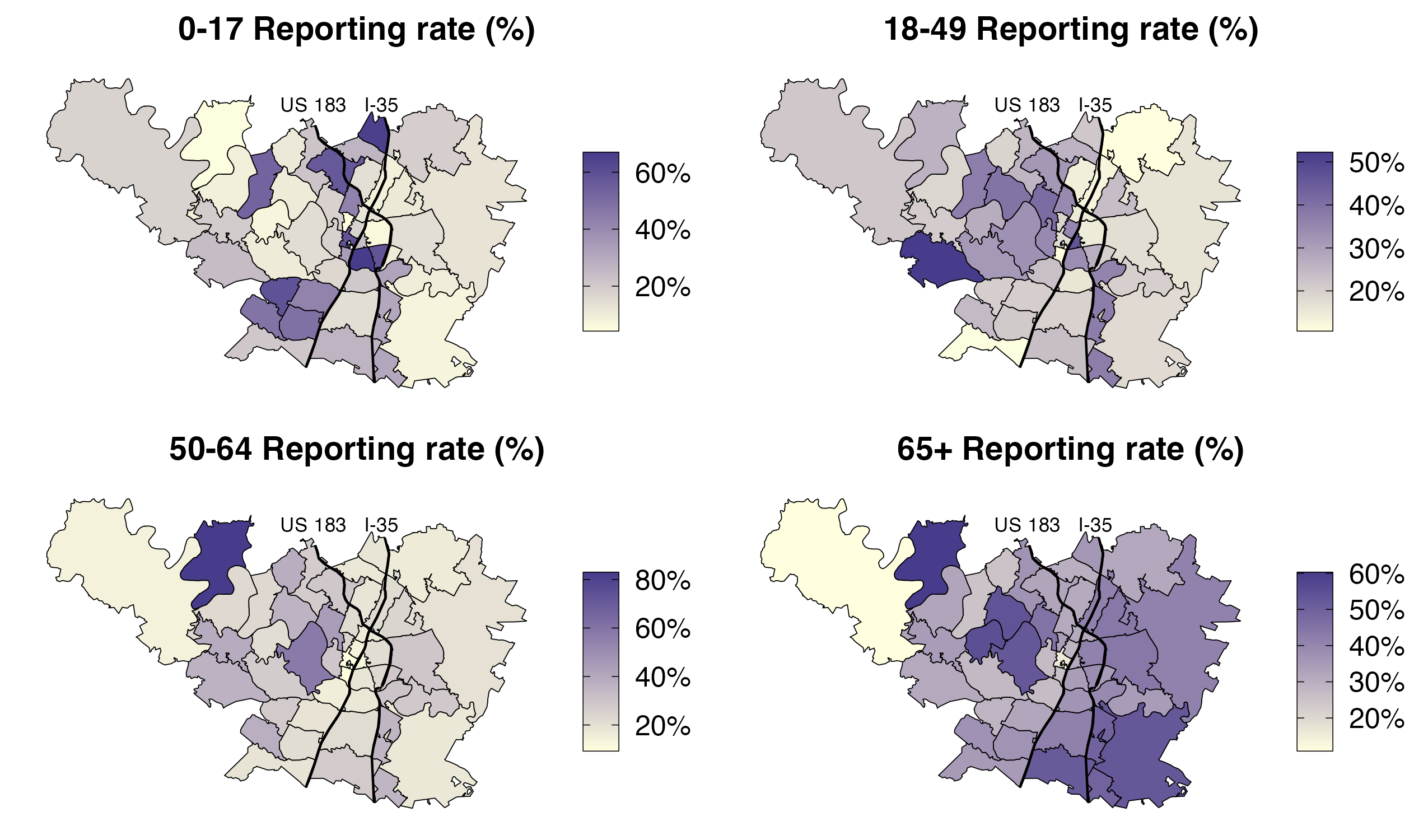

Supplement: S10 Fig — Testing data used for reporting rates are only a subset of all tests performed, as age and ZIP code stratified data were only available for Austin Public Health administered tests. (TIFF) [file pcbi.1011149.s010.tiff]

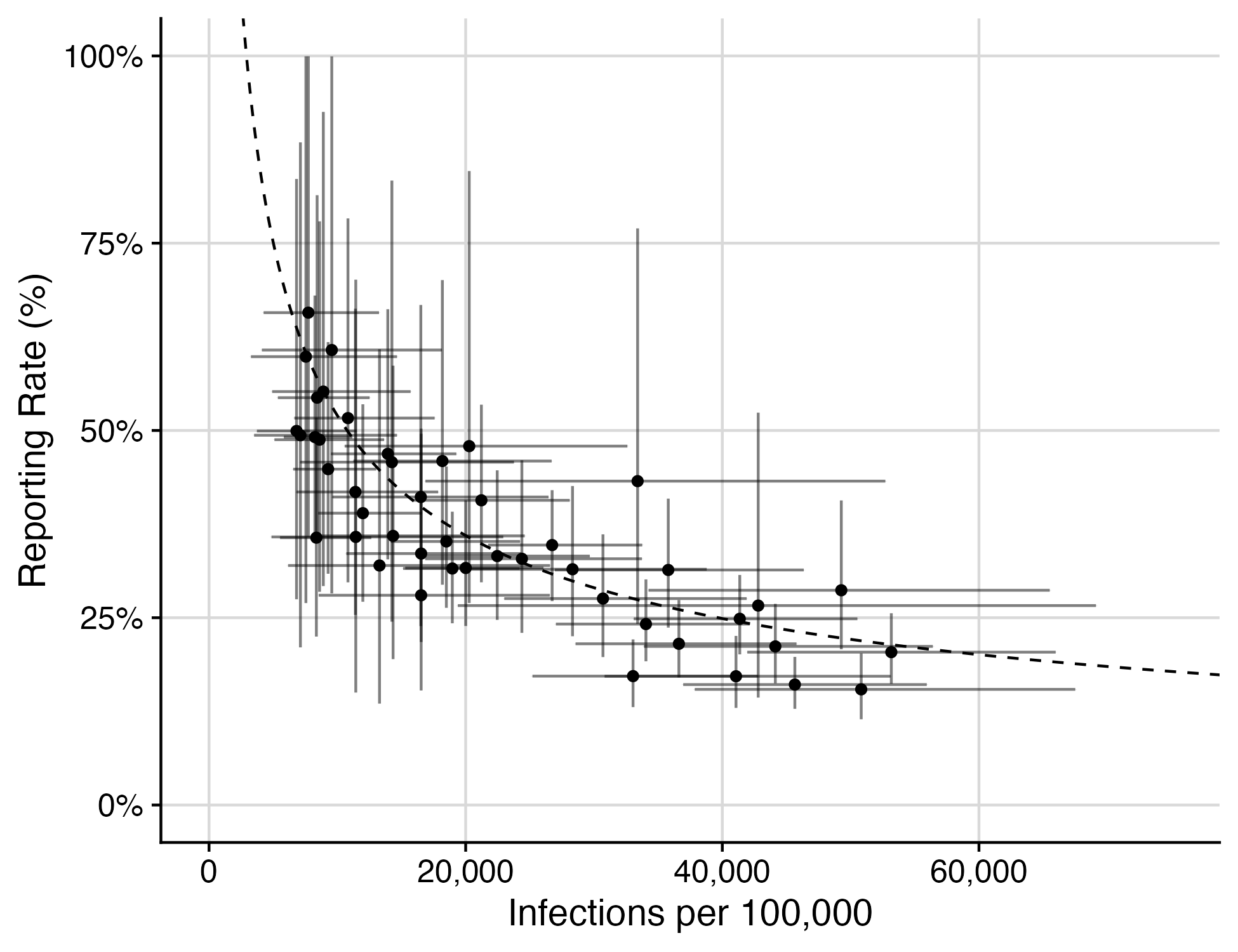

Supplement: S11 Fig — Points and error bars indicate the mean and 95% confidence intervals for each ZIP code. The dashed line indicates the mean estimated relationship across 1,000 posterior samples, with a = 70.4 (95% CrI: 28–141) and b = 0.533 (95% CrI: 0.45–0.61). (TIFF) [file pcbi.1011149.s011.tiff]

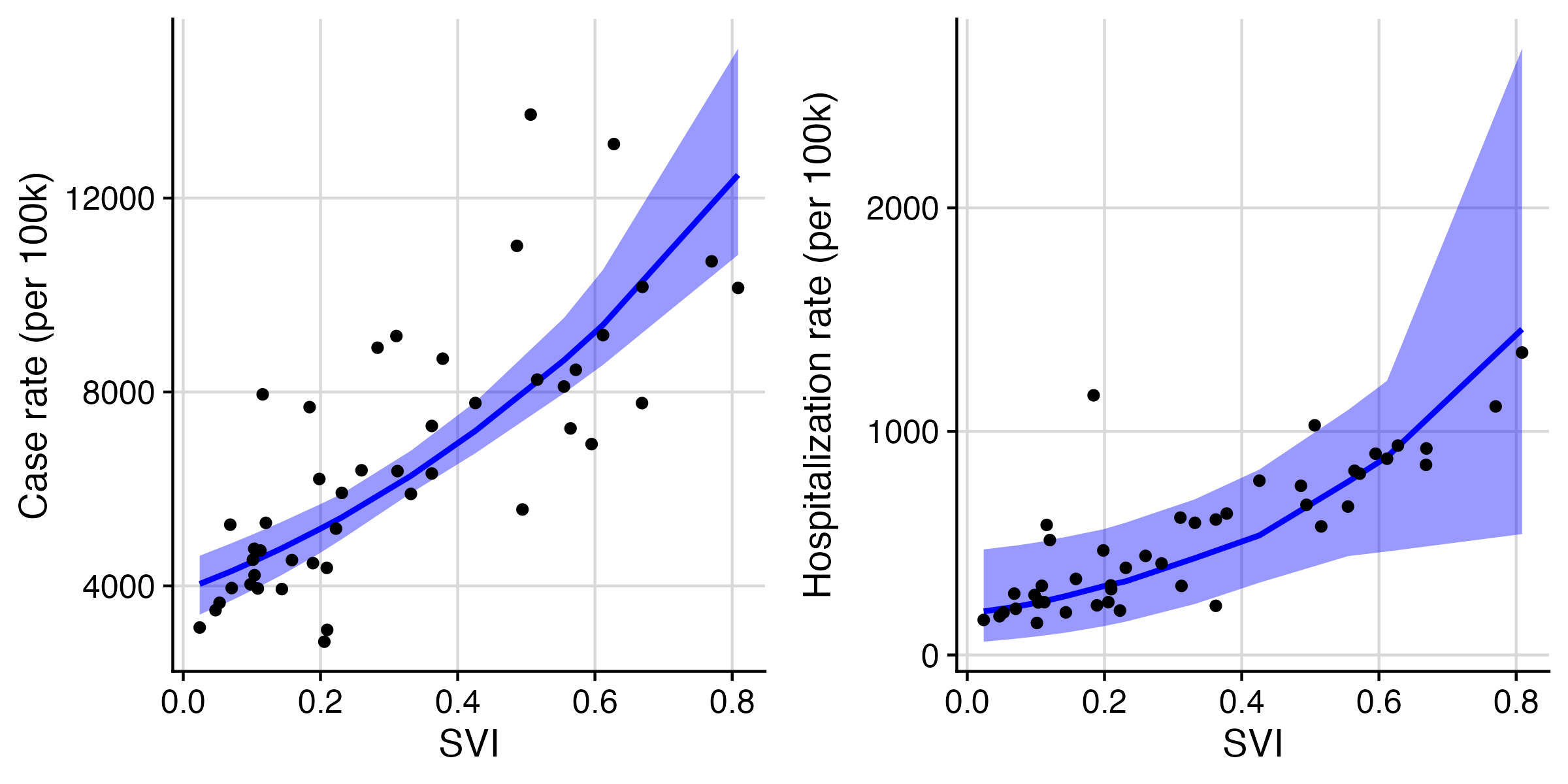

Supplement: S12 Fig — (A) Across the 46 ZIP codes, SVI is a significant predictor of reported case counts (p<0.001). The blue line and ribbon indicate the mean and 95% prediction interval from the fitted Poisson mixed-effects model. (B) Across the 46 ZIP codes, SVI is a significant predictor of reported hospitalization counts (p<0.001). The blue line and ribbon indicate the mean and 95% prediction interval from the fitted Poisson mixed-effects model. (TIFF) [file pcbi.1011149.s012.tiff]

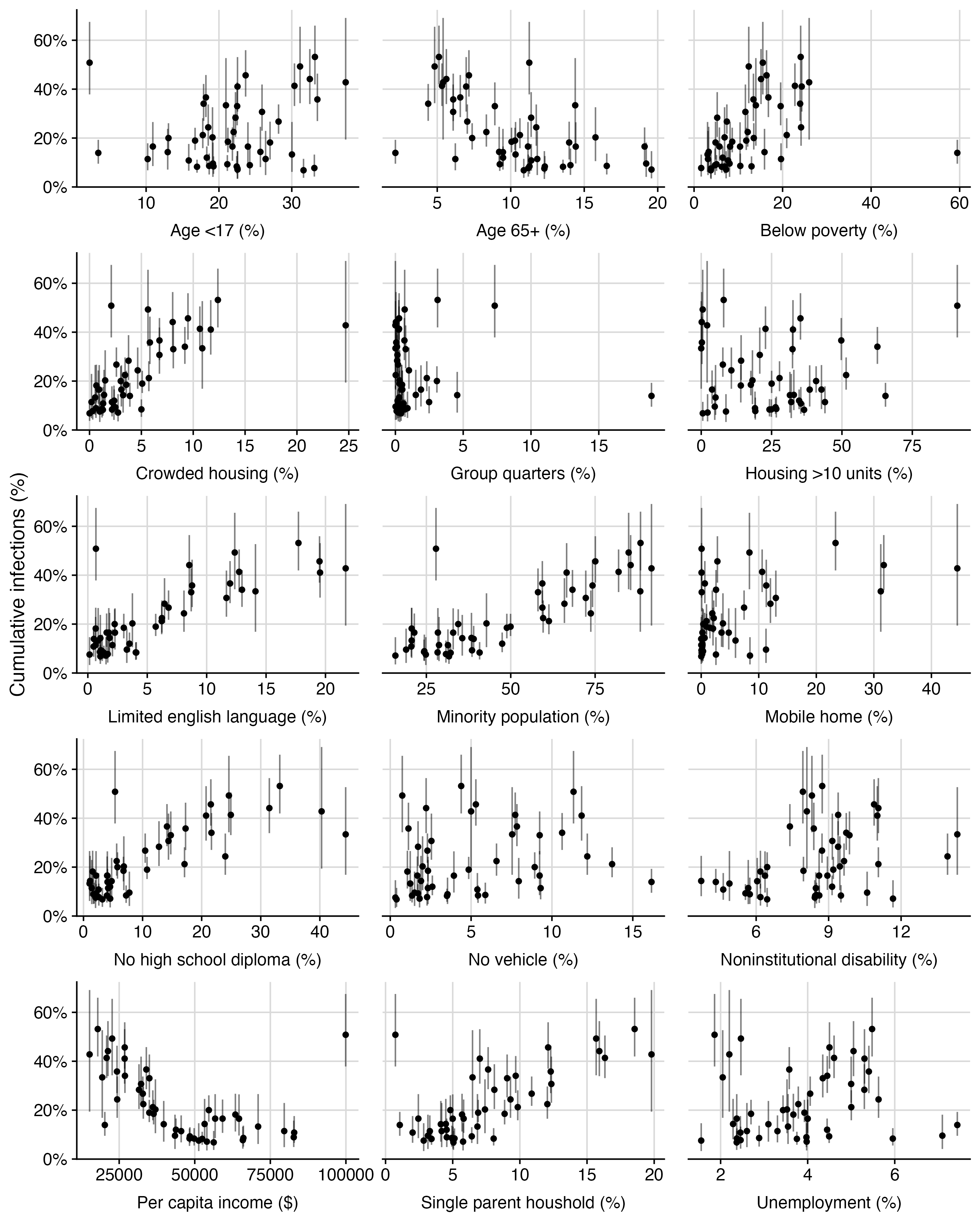

Supplement: S13 Fig — Points and error bars indicate the mean and 95% credible intervals for estimated infection rates in a ZIP code. (TIFF) [file pcbi.1011149.s013.tiff]

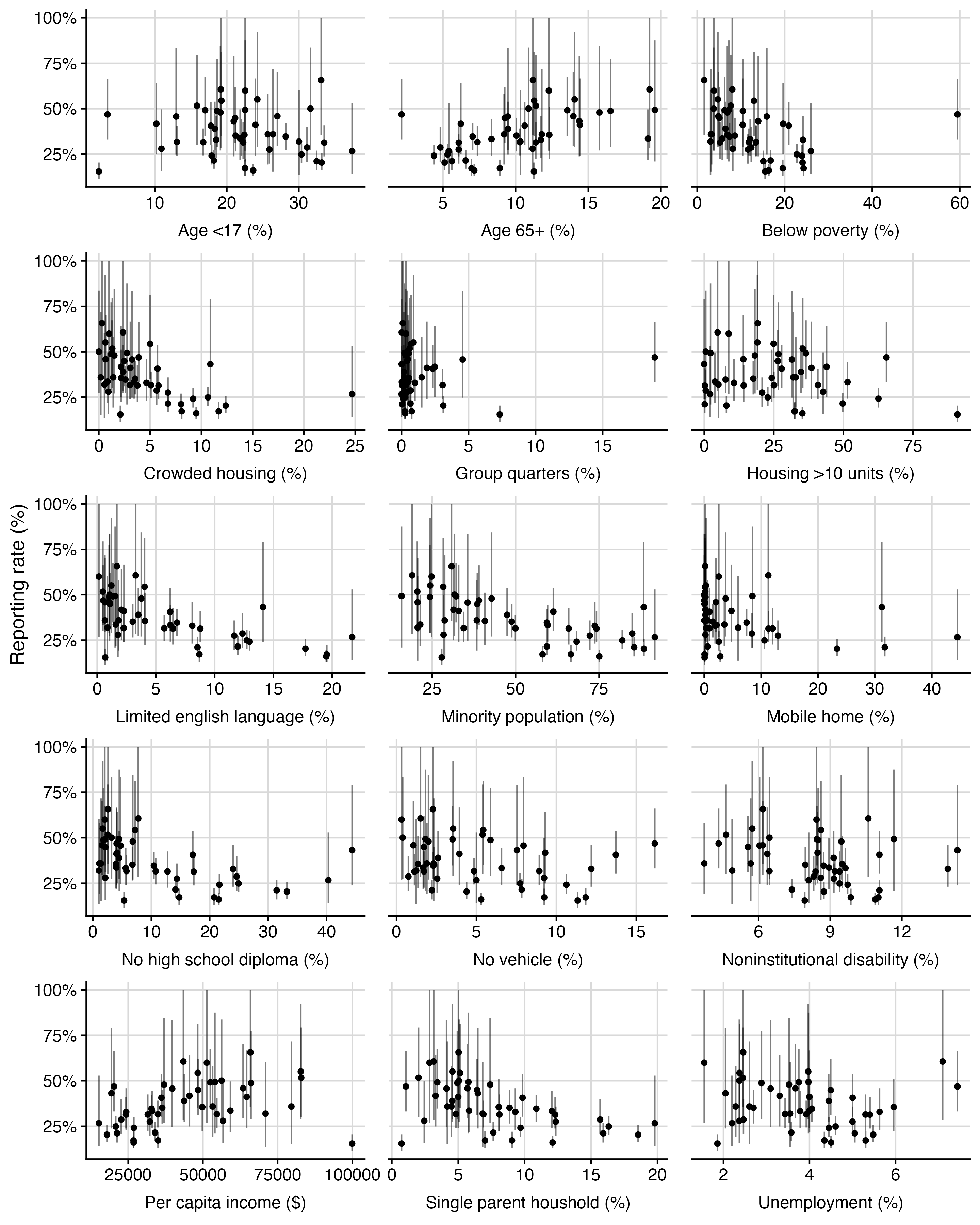

Supplement: S14 Fig — Points and error bars indicate the mean and 95% credible intervals for estimated reporting rates in a ZIP code. (TIFF) [file pcbi.1011149.s014.tiff]

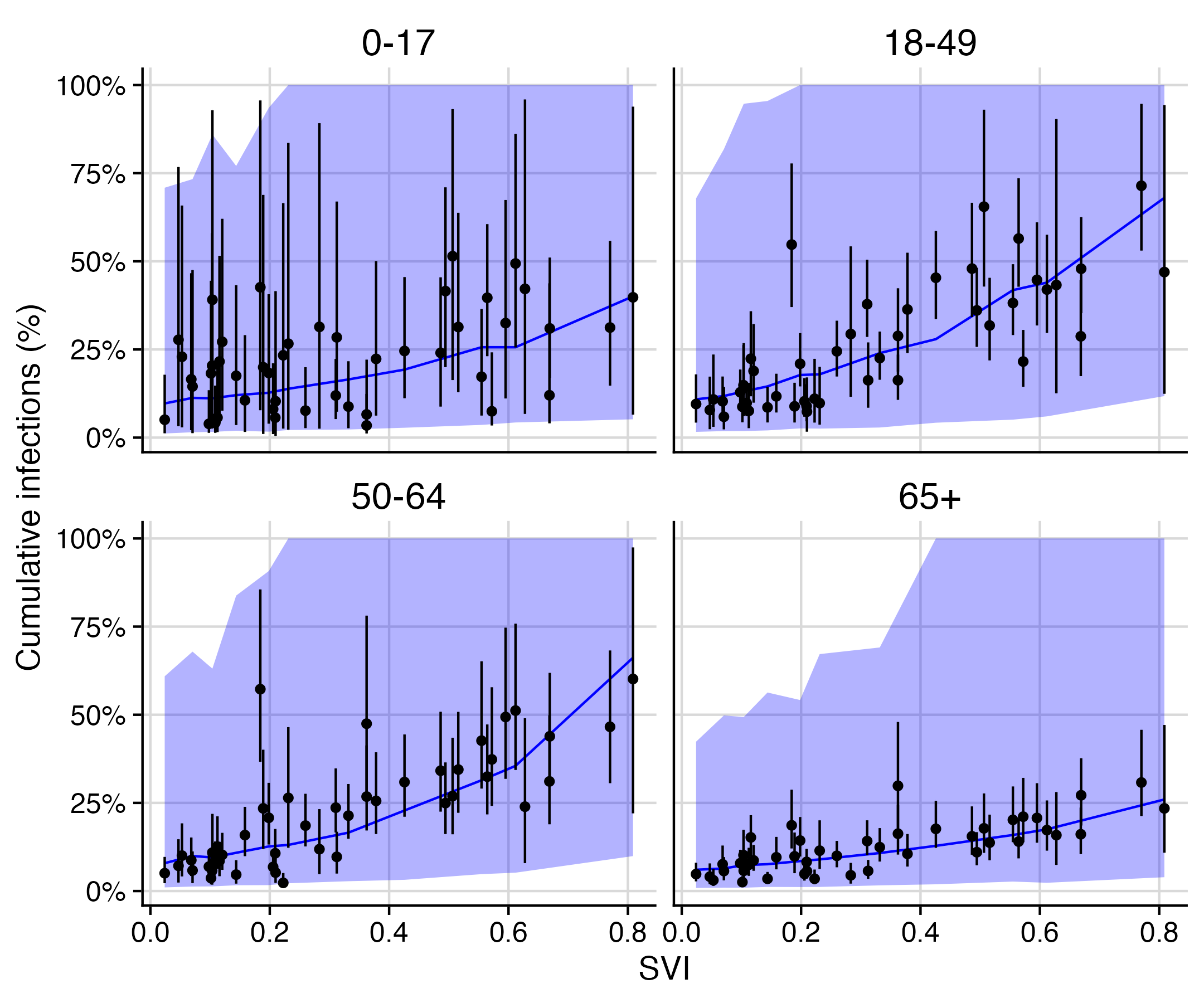

Supplement: S15 Fig — Across the 46 ZIP codes, SVI has a positive relationship with cumulative infection rates as a proportion of the population for every age group (S1 Table). Estimated age-specific SVI relationships from the poisson mixed effects regression model are shown in the blue line (mean) and blue ribbon (95% confidence interval). (TIFF) [file pcbi.1011149.s015.tiff]

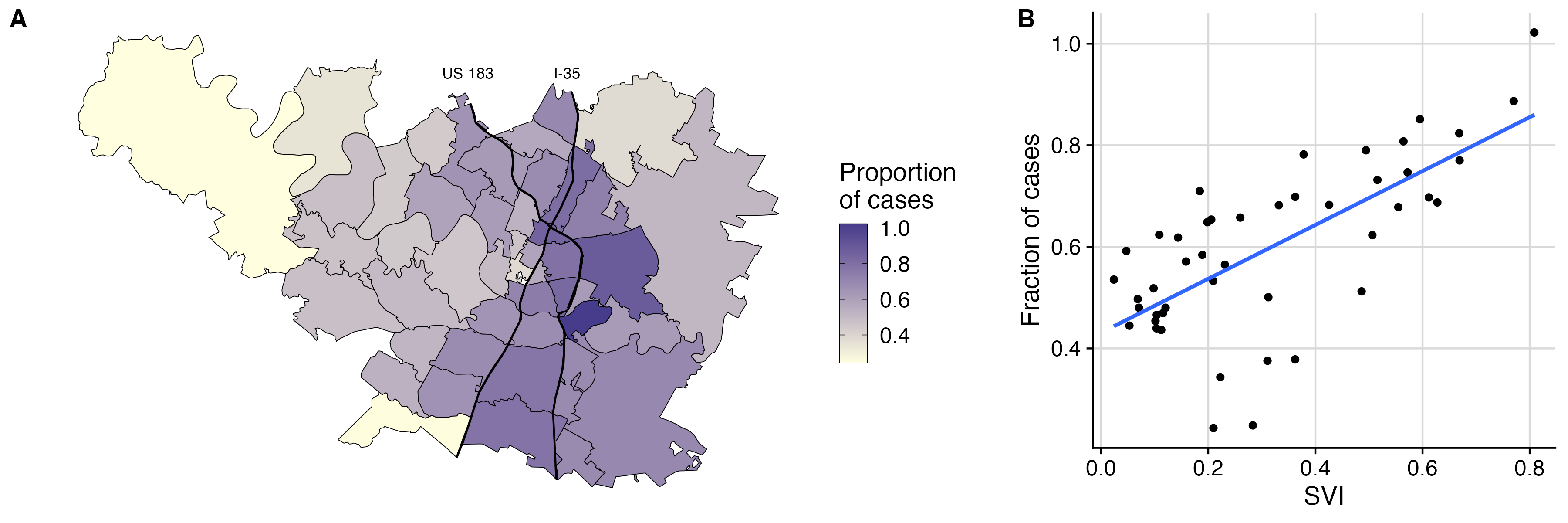

Supplement: S16 Fig — (A) Fraction of all reported cases included in the subset of age- and ZIP-code stratified data collected through Austin Public Health’s community testing programs by ZIP code. Overall, the data set covers 60% of all reported cases, but the data set, which does not include all cases identified by private testing sites, has high levels of coverage in the vulnerable ZIP codes of East Austin. (B) Reported case coverage from the dataset correlates positively with SVI. Blue line indicates the mean of a fitted linear regression model. (TIFF) [file pcbi.1011149.s016.tiff]

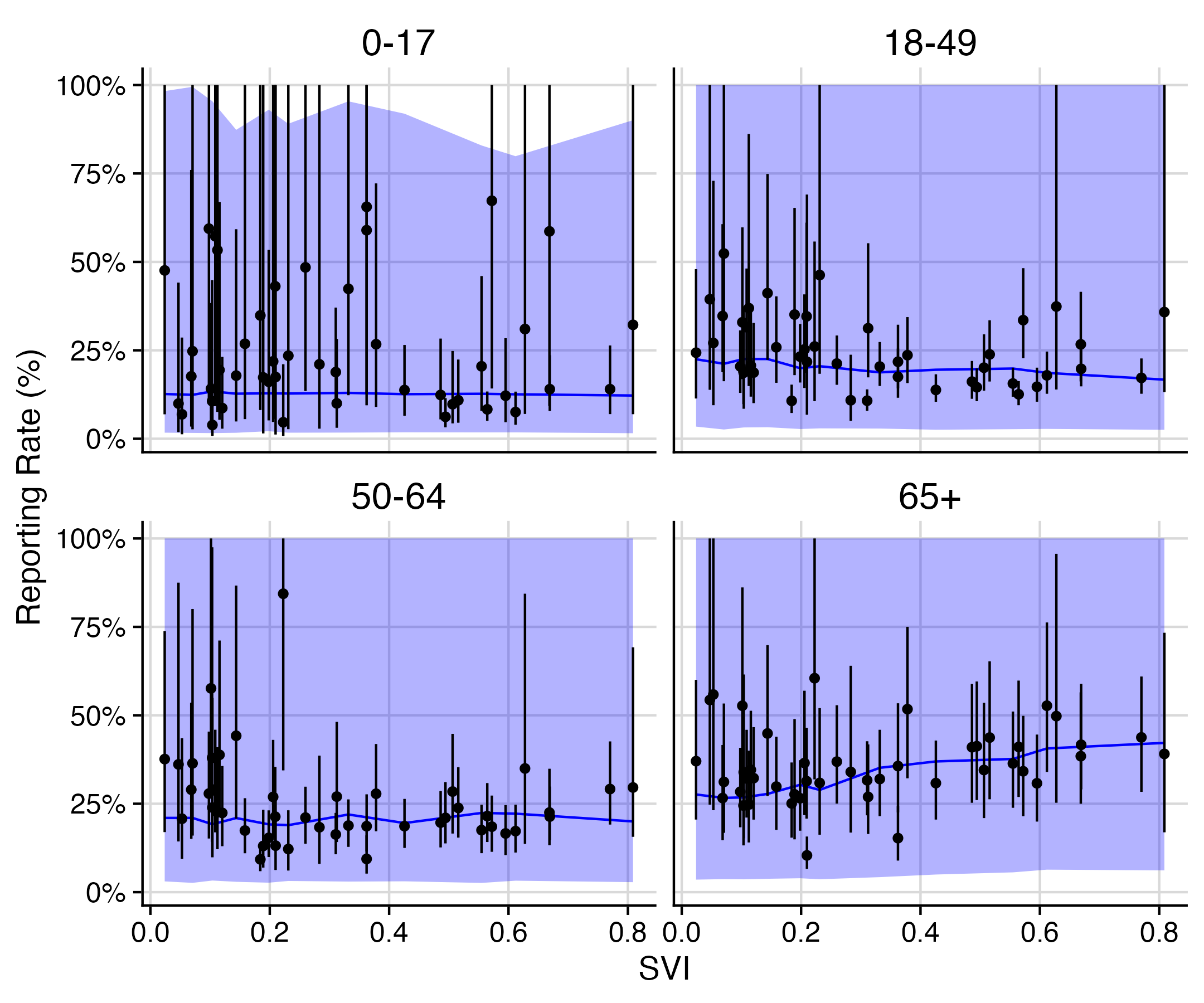

Supplement: S17 Fig — Across the 46 ZIP codes, SVI has a flat or slightly negative relationship with cumulative infection reporting rates for every age group except for those aged 65+ (S1 Table). Estimated age-specific SVI relationships from the poisson mixed effects regression model are shown in the blue line (mean) and blue ribbon (95% confidence interval). (TIFF) [file pcbi.1011149.s017.tiff]

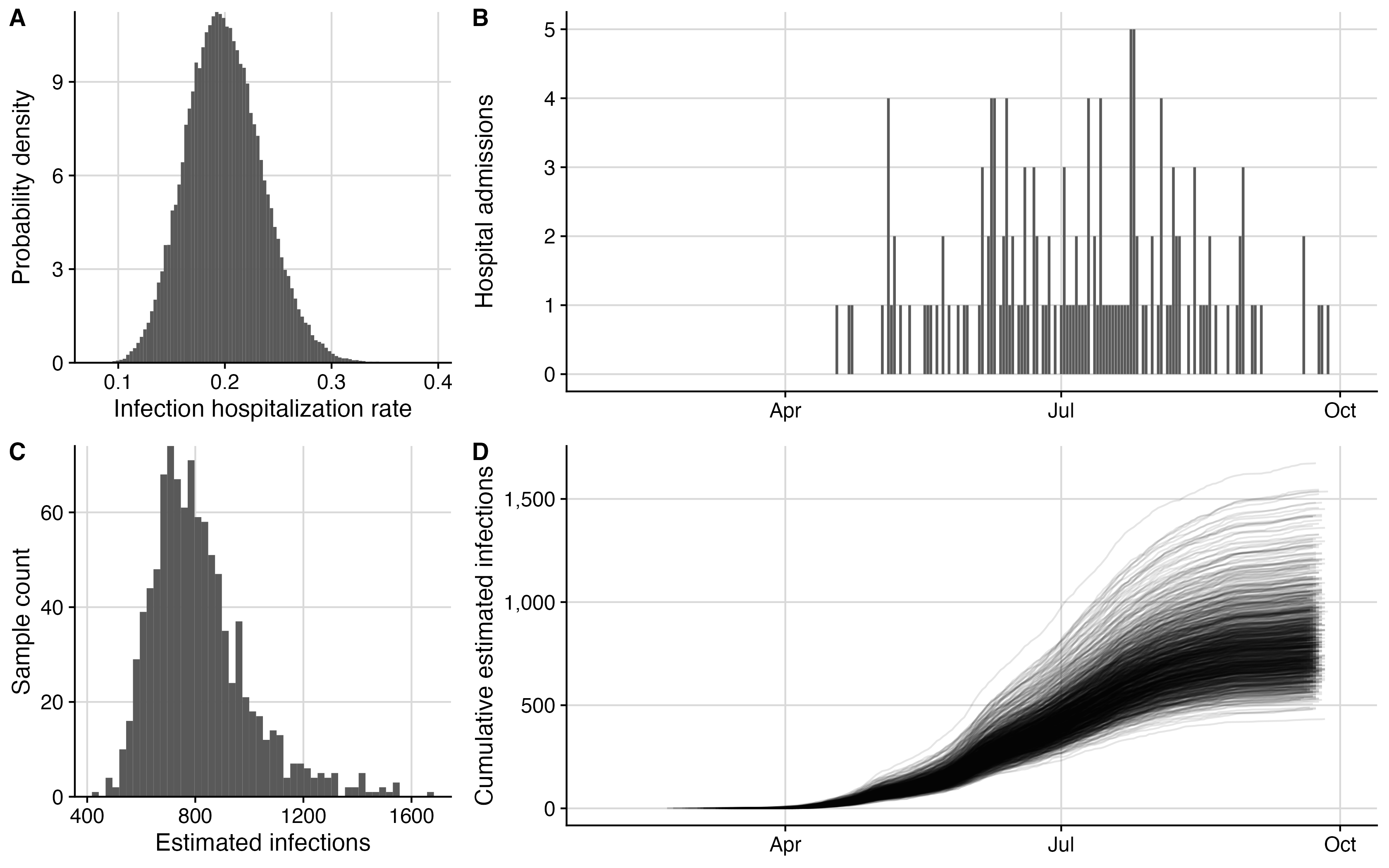

Supplement: S18 Fig — (A) Prior distribution of the infection hospitalization rate for the example region specified by α = 25 and β = 100. (B) Hospital admission counts by day in the example region. (C) Estimated cumulative infection distribution for the region based on the hospital admission count and IHR distribution. IHR distribution is made up of 1,000 draws from the posterior distribution. (D) Cumulative estimated infections over time for each of the 1,000 posterior infection draws. Timing is based on the hospital admission timing and the delay distribution between infection and hospitalization. (TIFF) [file pcbi.1011149.s018.tiff]
